# Müller Glial Kir4.1 Channel Dysfunction in *APOE4*‐KI Model of Alzheimer's Disease

**DOI:** 10.1002/glia.70119

**Published:** 2026-01-08

**Authors:** Surabhi D. Abhyankar, Yucheng Xiao, Neha Mahajan, Qianyi Luo, Theodore R. Cummins, Adrian L. Oblak, Bruce T. Lamb, Timothy W. Corson, Ashay D. Bhatwadekar

**Affiliations:** ^1^ Department of Ophthalmology Indiana University School of Medicine Indianapolis Indiana USA; ^2^ Department of Biochemistry and Molecular Biology Indiana University School of Medicine Indianapolis Indiana USA; ^3^ Department of Biology, School of Science Indiana University Indianapolis Indiana USA; ^4^ Stark Neurosciences Research Institute Indianapolis Indiana USA; ^5^ Leslie Dan Faculty of Pharmacy University of Toronto Toronto Ontario Canada

**Keywords:** *APOE*, Kir4.1, late‐onset Alzheimer's disease, mitochondrial dysfunction, Müller cells

## Abstract

Alzheimer's disease (AD), particularly late‐onset AD (LOAD), affects millions worldwide, with the apolipoprotein *ε*4 (*APOE4*) allele being a significant genetic risk factor. Retinal abnormalities are a hallmark of LOAD, and our recent study demonstrated significant age‐related retinal impairments in *APOE4*‐knock‐in (KI) mice, highlighting that retinal impairments occur before the onset of cognitive decline in these mice. Müller cells (MCs), key retinal glia, are vital for retinal health, and their dysfunction may contribute to retinal impairments seen in AD. MCs maintain potassium balance via specialized inwardly rectifying K^+^ channels 4.1 (Kir4.1). This study posits that Kir4.1 channels will be impaired in *APOE4*‐KI, resulting in MC dysfunction. Additionally, we demonstrate that MC dysfunction in *APOE4*‐KI stems from alterations in mitochondrial dynamics and oxidative stress. Kir4.1 expression and function were studied using immunofluorescence and through the whole‐cell voltage clamp, respectively. In parallel, rat Müller cells (rMC‐1) were used to create an in vitro model for further mechanistic studies. MitoQ was used to evaluate its potential to mitigate *APOE4*‐induced deficits. *APOE4* retinas and *APOE4*‐transfected rMC‐1 significantly reduced Kir4.1 expression, K+ buffering capacity, and increased mitochondrial damage. *APOE4*‐transfected rMC‐1 showed reduced mitochondrial membrane potential (ΔΨm) and increased mitochondrial reactive oxygen species (ROS). MitoQ treatment significantly reduced mitochondrial ROS and restored Kir4.1 expression in *APOE4*‐expressing cells. Our results demonstrate that *APOE4* causes mitochondrial dysfunction and MC impairment, which may contribute to retinal pathology in AD. MitoQ restored mitochondrial health and Kir4.1 expression in *APOE4*‐expressing rMC‐1, suggesting targeting mitochondria may offer a promising therapeutic strategy for AD.

## Graphical abstract

- *APOE4* impairs Müller cell health by reducing Kir4.1 expression and buffering.
- *APOE4* causes mitochondrial dysfunction with decreased ΔΨm and increased ROS.
- MitoQ restores Kir4.1 expression and reduces ROS in *APOE4*‐transfected cells.

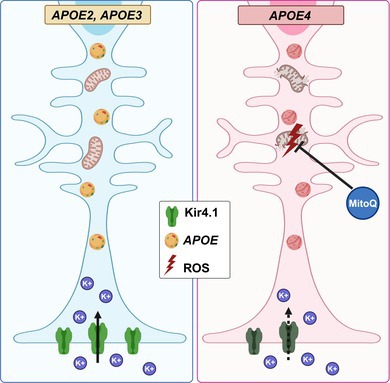

**Abbreviations**
*APOE2*
: apolipoprotein *E2*
*APOE3*
: apolipoprotein *E3*
*APOE4*
: apolipoprotein *E4*
EV: empty vector
Kir4.1: inwardly rectifying K^+^ channels 4.1
MC: Müller cell
MitoQ: mitoquinone mesylate
rMC‐1: rat Müller cell‐1
ΔΨm: mitochondrial membrane potential

## 1 Introduction

Over 55 million people worldwide are living with dementia, with Alzheimer's Disease (AD) being its most common form, responsible for roughly 60%–80% of cases globally (World Health Organization Alzheimer's Report 2021). In the United States alone, an estimated 6.7 million individuals aged 65 and older were diagnosed with AD in 2023. AD progressively impairs memory, learning, and executive functions (Bondi et al. 2017; Breijyeh and Karaman 2020), making it increasingly difficult for individuals to make decisions, solve problems, communicate, or care for themselves (Silva et al. 2019). The most prevalent subtype of AD is late‐onset AD (LOAD), which represents ~95% of all AD cases worldwide (Boutajangout and Wısnıewskı 2013) and affects nearly 30% of people over age 85 (Alzheimer΄s Association 2024). The *APOE4* allele is recognized as a significant genetic risk factor for LOAD (Uddin et al. 2018; Yamazaki et al. 2019), with 56% of AD patients in the United States carrying one copy of the *APOE4* allele and 11% carrying two copies (Alzheimer's Association 2023; Rajan et al. 2021). The three *APOE* gene variants *APOE2* (cystine 112, cystine 158), *APOE3* (cystine 112, arginine 158), and *APOE4* (arginine 112, arginine 158) have differing effects on AD risk: while the *APOE4* allele raises risk, the *APOE2* allele is considered protective, and the *APOE3* allele is neutral (Husain et al. 2021; Roses 1996).

The retina shares many characteristics with the brain, including vascular connections, neural pathways, and immune regulations, and it often mirrors brain pathology (Golzan et al. 2017; Lim et al. 2016; Patton et al. 2005). Recent studies from our group have shown that 52–57‐week‐old *APOE4*‐knock mice had retinal structural, functional, vascular, and vision deficits, increased neuroinflammation, and downregulation of synaptogenesis, suggesting middle‐aged *APOE4* mice have retinal dysfunction (Abhyankar et al. 2025). Müller cells (MCs), the most abundant retinal glial cells, span the retina and provide structural support to neurons (Kobat and Turgut 2020; Reichenbach and Bringmann 2013), akin to astrocytes in the brain, helping to maintain the blood‐retinal barrier by stimulating the production of tight junction proteins in endothelial cells (Bernardos et al. 2007). MC gliosis, a hallmark of AD‐related pathology, involves generalized and potentially protective responses, such as elevated glial fibrillary acidic protein (GFAP) and diminished glutamine synthetase (GS) levels (Bringmann et al. 2006). Prominent MC activation has been observed in several AD mouse models, including App^NL‐G‐F^, 5xFAD, and 3xTG, emphasizing its significance in disease progression (Edwards et al. 2014; Vandenabeele et al. 2021; Zhang et al. 2021). Consistent with these findings, decreased GS levels have been reported in the brains (Kulijewicz‐Nawrot et al. 2013; Le Prince et al. 1995; Olabarria et al. 2011; Robinson 2001) and the retinas of individuals with AD (Tams et al. 2022; Xu et al. 2022).

MCs perform crucial roles in neurotransmitter uptake, glycogen storage, and maintaining water and K^+^ balance (Bringmann et al. 2006; Kobat and Turgut 2020), largely through inwardly rectifying K^+^ channels 4.1 (Kir4.1) (Beverley and Pattnaik 2022; Bringmann et al. 2006). Kir4.1 channels help stabilize the retinal membrane potential and manage K^+^‐glutamate levels (Connors and Kofuji 2006; Katoozi et al. 2020; Li et al. 2021; Reichenbach and Bringmann 2013). Diabetes has been shown to decrease Kir4.1 expression, leading to MC swelling and altered Kir4.1 distribution (Luo et al. 2019), which compromises MC function and disrupts retinal physiology (Lassiale et al. 2016). Such dysregulation in Kir4.1 can increase neuronal hyperexcitability (Amaratunga et al. 1996; Nwaobi et al. 2016) and impair K^+^ buffering (Bringmann et al. 2006; Kofuji et al. 2002). In AD, reduced Kir4.1 expression has been observed in postmortem brain samples with amyloid accumulation and mouse models of AD, suggesting a link between Kir4.1 dysfunction and AD pathology (Wilcock et al. 2009).

MCs are vital for maintaining retinal function, and their dysfunction, driven by factors like *APOE4*, can significantly impact retinal health and contribute to disease progression. However, this hypothesis has yet to be examined in the context of AD and *APOE4*. In this study, we have used *APOE4*‐knock in (KI) mice and rat MC line (rMC‐1) expressing *APOE4* to investigate the effects of *APOE4* on MCs and Kir4.1 channels. We aim to understand how *APOE4* influences mitochondrial dynamics and other cellular functions critical to retinal and neural health.

## 2 Methods

### 2.1 Animals

The humanized *APOE*‐KI mice were created via gene targeting, in which the native mouse *Apoe* gene was replaced with the human *APOE3* or *APOE4* gene. These mice were developed by the Model Organism Development and Evaluation for Late‐Onset Alzheimer's Disease (MODEL‐AD) consortium. These mice were homozygous for either the *APOE3* (*3/3*) or *APOE4* (*4*/*4*) alleles. Hereafter, we will refer to them as *APOE3* mice and *APOE4* mice, respectively. The mice were housed at the animal care facility of the Eugene and Marilyn Glick Eye Institute, Indiana University, Indianapolis, IN, USA. All the animals were maintained under standard physiological conditions, including a 12‐h light/dark cycle, with continuous access to food and water. All experiments followed the Guiding Principles in the Care and Use of Animals (National Institutes of Health) and the Association for Research in Vision and Ophthalmology (ARVO) Statement for the Use of Animals in Ophthalmic and Vision Research. Experiments were conducted on male and female animals (we did not observe any sex‐specific differences in the examined outcomes, and therefore the data were pooled) aged between 52 and 57 weeks of age.

### 2.2 Whole‐Cell Voltage‐Clamp Recording

At 52–57 weeks of age, mice were euthanized, and after the eyes were enucleated, the retinas were isolated. The retinas were then incubated in Ringer's solution containing 0.3 mg/mL papain and 2.5 mM L‐cysteine for 30 min at 37°C. Following this, the retinas were briefly incubated in Dulbecco's Modified Eagle's Medium (DMEM, Thermo Fisher Scientific, MA, USA) with 10% fetal bovine serum (FBS, Thermo Fisher Scientific, MA, USA) and 0.2 mg/mL DNase‐1 at room temperature (RT), and the tissue was gently triturated. The resulting cell suspension was layered over a discontinuous Percoll gradient (10%, 20%, 30%, and 50% Percoll) and centrifuged at 800 g for 5 min. The fraction enriched in MCs, found at the top of the 30% Percoll layer, was collected, washed with DMEM containing 10% FBS, and transferred to Poly‐L‐Lysine and laminin‐coated coverslips to promote cell adhesion.

Whole‐cell voltage‐clamp recordings were conducted at RT (~21°C) using an EPC‐10 amplifier and the Pulse program (HEKA Electronics, Lambrecht [Pfalz], DE). Fire‐polished electrodes (3.0–5.0 MΩ) were fabricated from 1.7 mm capillary glass using a P‐1000 puller (Sutter Instruments). The pipette solution consisted of 140 mM KF, 1.1 mM EGTA, 10 mM NaCl, and 10 mM HEPES (pH 7.3 with KOH). The bathing solution was 140 mM NaCl, 1 mM MgCl_2_, 3 mM KCl, 1 mM CaCl_2_, and 10 mM HEPES (pH 7.3 with NaOH) with and without 1 mM BaCl_2_. The offset potential was zeroed before contacting the cell. Once a whole‐cell recording configuration was established, the cell was held at −60 mV for 3 min to allow for intracellular equilibration. A family of Kir currents was induced by a 50‐ms step pulse, ranging from −140 to +30 mV in 10‐mV increments. Voltage errors were minimized by compensating for 50%–70% of the series resistance. The capacitance artifact was canceled using the computer‐controlled circuitry of the patch‐clamp amplifier, but no linear leak subtraction was performed. Membrane currents were typically filtered at 5 kHz and sampled at 20 kHz.

### 2.3 Cell Culture and Transfections

rMC‐1 was generously provided by Dr. Vijay Sarthy, Northwestern University, Chicago, IL, USA. The cells were cultured in low glucose, no phenol red, DMEM (Thermo Fisher Scientific, MA, USA) supplemented with 10% FBS, 1% L‐glutamine (Corning, VA, USA), and 1% antibiotic‐antimycotic (Thermo Fisher Scientific, MA, USA). rMC‐1 was grown in DMEM overnight and transfected with 1 μg of plasmids encoding human *APOE* isoforms: pCMV4‐*APOE2* (Cat. #87085, addgene, MA, USA), pCMV4‐*APOE3* (Cat. #87086, addgene), and pCMV4‐*APOE4* (Cat. #87087, addgene). Cells transfected with empty vector (EV, pCMV4‐HA, Cat. #27553, Addgene) were used as a control. The human APOE plasmids do not have any tag, while the EV has an HA tag. Transfections were performed using Lipofectamine 3000 (L3000‐008, Invitrogen, Thermo Fisher Scientific, MA, USA) as per the manufacturer's protocol, with the transfection efficacy of ~70%–80%. Cells were collected 24 h post‐transfection for mRNA, protein, and flow cytometry analyses. The validation of transfection was performed using immunofluorescence staining.

### 2.4 Immunofluorescence

At 52–57 weeks of age, mice were euthanized, and their eyes were fixed in 4% Paraformaldehyde (PFA) solution for 15 min at RT, followed by rinsing with phosphate‐buffered saline (PBS). The retinas were then isolated from the fixed eyes, embedded in 3% agarose, and sectioned with a vibratome. Agarose sections were washed in a buffer containing 3% dimethyl sulfoxide (DMSO, Thermo Fisher Scientific, MA, USA) and 0.3% TritonX‐100 (Thermo Fisher Scientific, MA, USA) in PBS, then blocked for 2 h at RT with 5% goat serum diluted in washing buffer. Sections were then incubated overnight at 4°C with primary antibodies, including Kir4.1 (Cat. #APC‐035‐GP, Alomone Labs, 1:200), glutamine synthetase (GS, Cat. #MAB302, Millipore, 1:200), TOMM20 (Cat. #MA5‐32148, Invitrogen, 1:100) and Aquaporin 4 (AQP4, Cat. #sc‐ 32,739, Santa Cruz Biotechnology Inc., TX, USA, 1:100). The next day, sections were incubated with appropriate secondary antibodies. To validate transfections, rMC‐1 was seeded on an 8‐well chamber slide and transfected as described earlier. Later, cells were fixed with 4% PFA for 15 min, permeabilized using 0.3% TritonX‐100 diluted in PBS, and blocked for 1 h with 5% goat serum diluted in permeabilization solution. The cells were then incubated O/N at 4°C with Anti‐HA (Cat. #26183, Invitrogen, 1:200), APOE (Cat. #ab52607, Abcam, 1:100), APOE3 (Cat. #MAB41442‐SP, Novus Biologicals, CO, USA, 1:100) and APOE4 (Cat. #NBP1‐49529SS, Novus Biologicals, 1:100) antibody, followed by washing and a 2‐h incubation with the appropriate secondary antibody the next day. Transfected rMC‐1 were stained with TOMM20 to check the effect of *APOE* isoforms on mitochondria. To check whether cholesterol/lipid levels affect Kir4.1, we stained retinal sections as well as rMC‐1 with Kir4.1 and BODIPY (Cat. #D3922, Thermo Fisher Scientific, 1:2000). Following secondary antibody incubation, BODIPY was added to retinal sections and rMC‐1 and incubated for 1 h at RT, followed by mounting. Images were captured using a Zeiss LSM‐700 confocal microscope (Carl Zeiss MicroImaging, Germany). The fluorescence intensities from the retinal sections for Kir4.1, TOMM20, and GS were calculated by subtracting fluorescence intensity from the secondary antibody control. The integrated density per cell area for TOMM20 staining in rMC‐1 was calculated from total Z‐stack projections using Fiji ImageJ software.

### 2.5 qRT‐PCR for mRNA Analysis

Total RNA was extracted using Trizol reagent (Thermo Fisher Scientific, MA, USA) following the manufacturer's instructions, and 1 μg of RNA was then reverse‐transcribed with the SuperScript VILO cDNA synthesis kit (Thermo Fisher Scientific, MA, USA). Quantitative real‐time PCR was performed using gene‐specific primers, TaqMan Fast Universal Master Mix (Thermo Fisher Scientific, MA, USA), and the Viia7 Real‐Time PCR system (Thermo Fisher Scientific, MA, USA) to measure mRNA levels. mRNA expression levels for each gene were normalized to the housekeeping gene *Bact* (Rn00667869_m1). Primers used were *Kcnj10* (gene for *Kir4.1*; Rn00581058_m1), *Mfn1* (gene for Mitofusin‐1, Rn00594496_m1), *Mfn2* (gene for Mitofusin‐2, Rn00500120_m1), and *Dnm1* (gene for Dynamin‐1, Rn00586466_m1).

### 2.6 Pathway Analysis of RNA Sequencing Data

We performed additional pathway analysis on mRNA sequencing of the retinas of *APOE3* and *APOE4* mice from our previously published reports (Abhyankar et al. 2025) using the Ingenuity Pathway Analysis (IPA). Briefly, the gene targets obtained from the sequencing data were uploaded to IPA software, followed by expression analysis using the “core analysis” feature. The expression *p* value cut‐off was set to < 0.05, and both direct and indirect relationships were allowed in the pathway analysis. For pathway analysis, we used the “pathway designer” function of IPA, where all the desired targets were added, and using the ‘grow’ function, the possible networks were grown onto individual targets. Finally, these targets overlapped with transcriptomics data to include the gene targets and exclude the predicted mRNA targets not found in our sequencing data.

### 2.7 Western Blotting

RIPA buffer (#R0278, Sigma‐Aldrich Corp.) containing a protease inhibitor mixture was used to lyse rMC‐1. Protein concentrations were measured using the BCA assay (Pierce, Thermo Fisher Scientific), and equal amounts of protein were loaded onto a 4%–12% Bis‐Tris gel (Novex, Thermo Fisher Scientific) for separation. Proteins were then transferred onto a PVDF membrane and blocked with 4% BSA in TBST buffer. The membranes were probed with primary antibodies against α‐Tubulin (Cat. #T9026, 1:2000; Sigma‐Aldrich Corp.) and Kir4.1 (Cat. # APC‐035, 1:2000, Alomone Labs) O/N at 4°C. The next day, the membranes were incubated with secondary peroxidase antibodies at RT for 2 h. Bands were visualized using an ECL2 western blotting substrate (Thermo Fisher Scientific) and scanned with a Typhoon FLA 9500 laser scanner (GE Healthcare Life Sciences, PA, USA). Protein band intensities were quantified using ImageJ software. Integrated optical density (IOD) was calculated by taking the ratio of Kir4.1 and α‐tubulin.

### 2.8 Seahorse XF24 Metabolic Flux Assay

For Seahorse XF24 metabolic flux assay, 25,000 rMC‐1 were seeded per well into Seahorse XF24 microplates and allowed to adhere for 24 h. rMC‐1 were maintained in low‐glucose DMEM with 10% FBS. The following day, cells were transfected with either *EV*/*APOE2*/*APOE3*/*APOE4* and allowed to grow for another 24 h. The next day, tissue culture media were replaced with Seahorse assay media (Agilent Technologies, Santa Clara, CA, USA) containing Seahorse XF DMEM, 25 mM glucose, 1 mM pyruvate, and 4 mM L‐glutamine, and cells were incubated for at least 1 h at 37°C in a non‐CO_2_ incubator in Seahorse assay medium before starting the assay. Metabolic analysis was performed using Agilent Seahorse XF24 analyzer and Mito stress test kit (Agilent Technologies) following the manufacturer's instructions. Three independent experiments were conducted, including three to four technical replicates per condition. Oxygen consumption rate (OCR) and extracellular acidification rate (ECAR) were recorded, followed by sequential injections of oligomycin (Oligo, 2 μM), FCCP (1 μM), and rotenone/antimycin A (Rot/AA, 0.5 μM). Proton production rate (PPR) was derived from ECAR and OCR values using Agilent's Seahorse Wave Desktop Software, providing an estimate of proton flux related to ATP production. OCR, ECAR, and PPR directly measured mitochondrial respiration, glycolysis, and total proton production, offering a quantitative, non‐probe‐based approach to assess bioenergetics.

### 2.9 Mitochondrial Membrane Potential (ΔΨm)

Twenty‐four hours after transfections, rMC‐1 were resuspended in 1 mL of DMEM at ~1 × 10^6^ cells/mL and incubated with 2 μM JC‐1 (5′,6,6′‐tetrachloro‐1,1′,3,3′‐tetraethylbenzimidazolylcarbocyanineiodide, Molecular Probes, Invitrogen, CA, USA) for 30 min at 37°C in the dark, following the manufacturer's instructions. Unstained and EV‐treated cells were used as a control. For each sample, 100,000 gated events were acquired using a BD LSR Fortessa cell analyzer (BD Biosciences, San Jose, CA, USA) with 582/15 nm (PE) filters for JC‐1 aggregates and 525/50 nm (FITC) filters for JC‐1 monomers. Data were analyzed using FlowJo software (TreeStar, OR, USA). Dead cells and debris were excluded based on forward and side scatter, and all analyses were gated on unstained cells based on forward and side scatter morphology.

### 2.10 Mitochondrial Reactive Oxygen Species (ROS) Measurement

rMC‐1 were incubated with 1 μM MitoSox Red (MSR) mitochondrial superoxide indicator (Molecular Probes, Invitrogen, CA, USA) for 30 min at 37°C in the dark, according to the manufacturer's instructions. Unstained and EV‐transfected cells were used as controls. For each sample, 100,000 gated events were recorded on a BD LSRFortessa (BD Biosciences, NJ, USA) cell analyzer using a 610/20 nm (PE‐Texas Red) filter. Data analysis was conducted using FlowJo v10.10 software (BD Life Sciences, NJ, USA). Dead cells and debris were excluded based on forward and side scatter, and analyses were gated on unstained cells.

### 2.11 Mitoquinone Mesylate (MitoQ) Treatment

A sample of 1 μM MitoQ (Cat. #317102, MedKoo Biosciences Inc., NC, USA) was prepared according to the manufacturer's instructions. To find the optimal dose of MitoQ, we made three concentrations of MitoQ: 0.5 μM, 1 μM, and 2 μM. 24 h after transfections, rMC‐1 was washed twice with PBS and incubated in serum‐free medium (SFM) for 2 h before MitoQ treatments. A 1:1 ethanol‐to‐water mixture was used as a vehicle. Cells were then treated with different concentrations of MitoQ and vehicle and incubated for 24 h at 37°C. After 24 h of treatment, gene expression of Kir4.1 was measured using qRT‐PCR as described previously. All the remaining experiments, such as western blot and MSR flow cytometry, were performed with 1 μM of MitoQ as described earlier.

### 2.12 Alamar Blue Viability Assay

rMC‐1 were seeded in a flat, clear bottom 96 well plate at a density of 25,000 cells/well in 100 μL DMEM, and transfections were carried out as mentioned previously. The following day, transfected cells were treated with MitoQ and vehicle, while non‐transfected cells received a 20% DMSO treatment as a positive control. After 24 h of MitoQ treatment, the medium was replaced with 100 μL SFM, and 11.1 μL of Alamar Blue (Bio‐Rad, CA, USA) was added to each well. Cells were incubated with Alamar Blue for 4 h at 37°C. Fluorescence was measured using a Synergy H1 plate reader (BioTek, Winooski, VT) with an excitation wavelength of 560 nm and an emission wavelength of 590 nm. Raw fluorescence values were normalized to the fluorescence of DMSO‐treated control cells. The % viable cells were calculated by taking a ratio of MitoQ‐treated cells and vehicle‐treated cells.

### 2.13 Casein Kinase Inhibitor (CK) Treatment

Samples of 200 nM CK1 and 5 μM CK2 (Cat. #7979 and #2275 respectively, Bio‐Techne, MN, USA) were prepared according to the manufacturer's instructions. Twenty‐four hours after transfections, rMC‐1 were washed twice with PBS and incubated in SFM for 2 h before CK1/CK2 treatments. DMSO was used as a vehicle. Cells were then treated with 200 nM CK1 and 5 μM CK2 inhibitors and vehicle and incubated for 24 h at 37°C. After 24 h of treatment, protein expression of Kir4.1 was measured using Western blot as described earlier.

### 2.14 Statistical Analysis

For the animal studies, we used *n* = 10 *APOE3* mice (31 cells), *n* = 9 *APOE4* mice (38 cells), for whole‐cell voltage‐clamp recording and *n* = 3 animals per group for immunofluorescence staining. For in vitro experiments, *n* = 3–5 independent experiments were performed with 3 technical replicates per experiment. Data were expressed as mean ± standard error of the mean (SEM) and analyzed with GraphPad Prism 10.0.1 for Windows (San Diego, California; www.graphpad.com). A *t*‐test was used to compare fluorescence intensities and current densities for whole‐cell voltage‐clamp recording. Intergroup comparisons were conducted using one‐way ANOVA followed by Tukey's multiple comparison test. A *p* value of less than 0.05 was considered statistically significant. *p < 0.05, **p < 0.01, ***p < 0.001, and ****p < 0.0001.

## 3 Results

### 3.1 *APOE4* Allele Causes Structural and Functional Deficits in the MCs

**FIGURE 1:**
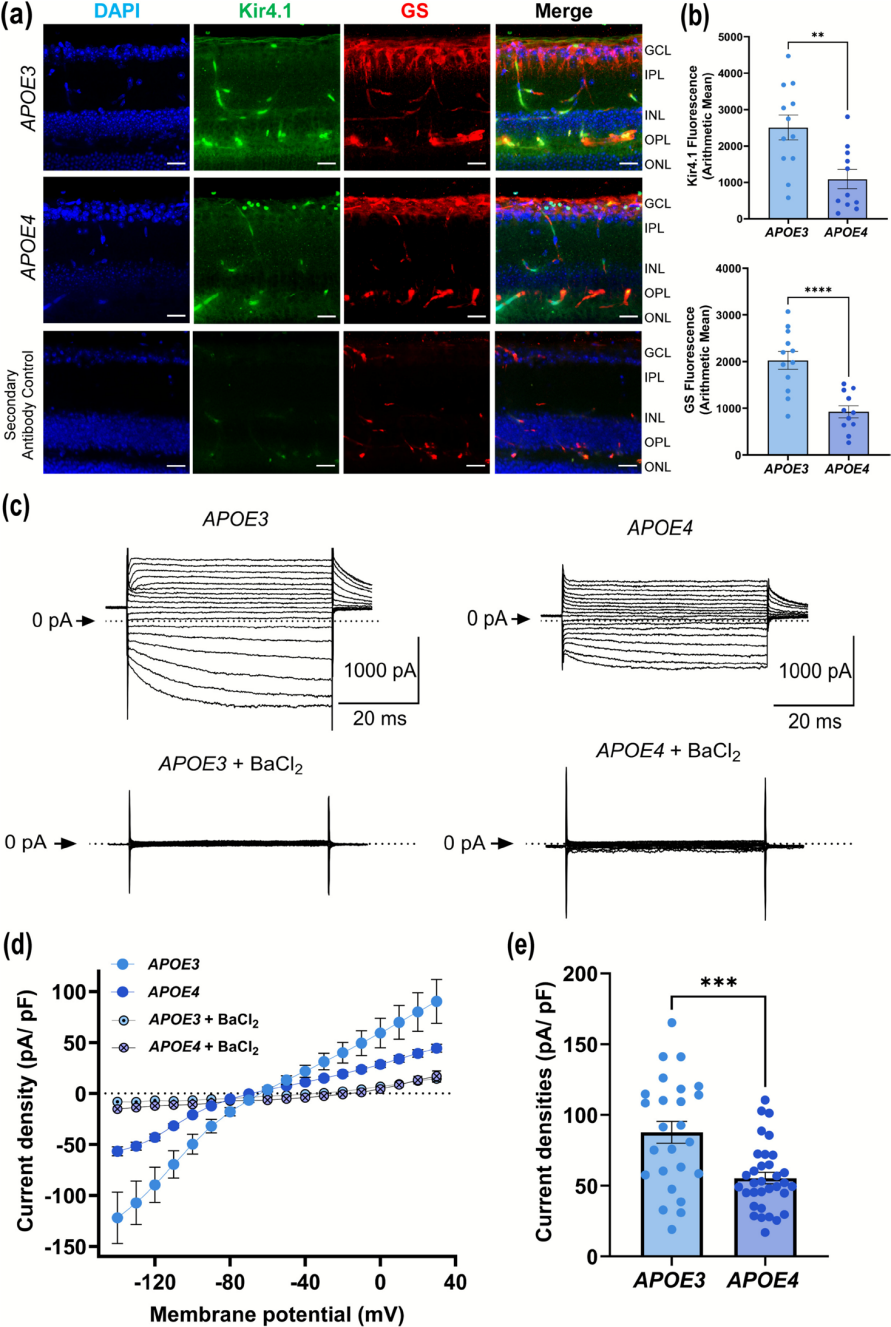
*APOE4* causes deficits in the Kir4.1. (a) Representative images of retinal slices showing Glutamine synthase (GS) and Kir4.1 staining pattern in *APOE3* and *APOE4* mice, scale 20 μm (*n*: *APOE3* = 3, *APOE4* = 3). (b) Bar graph showing quantification of immunofluorescence for Kir4.1 and GS (*n*: 11–12 images/group). (c) Representative current traces of Kir4.1 from freshly isolated Müller cells from *APOE3* and *APOE4* mice with and without 1 mM BaCl_2_ treatment. Currents were elicited by a 50‐ms hyperpolarization to −140 mV from a holding potential of −60 mV. The dashed line indicates the closed state (zero current), the downward pulses represent channel openings, corresponding to inward K^+^ current. The flickers indicate channel opening and closing. (d) Representative current–voltage (I–V) relationship of whole‐cell voltage‐gated K^+^ currents of Kir4.1 from freshly isolated Müller cells from *APOE3* and *APOE4* mice with and without 1 mM BaCl_2_ treatment. (e) Current densities of Kir4.1 from freshly isolated Müller cells from *APOE3* and *APOE4* mice collected from +30 mV (*n*: *APOE3* = 26 cells/9 mice, *APOE4* = 33 cells/8 mice). Values are expressed as mean ± SEM. An unpaired *t*‐test was used for statistical analysis. **p < 0.01, ***p < 0.001, ****p < 0.0001.

To study the effect of *APOE4* on MCs, agarose‐embedded retinal sections were stained for Kir4.1 and GS (Figure 1a). Kir4.1 is localized to the vitreal border and in perivascular processes in the outer retina (Connors and Kofuji 2006). *APOE4* retinas show minimal Kir4.1 expression in the vitreal border compared to the *APOE3* retinas. There was a marked reduction in both Kir4.1 (*p* = 0.0019) and GS (*p* < 0.0001) in *APOE4* retinas compared to the *APOE3* retinas (Figure 1b), suggesting impaired structural integrity in MCs is associated with the *APOE4* allele. To further assess Kir4.1 function, we conducted whole‐cell patch clamp recordings in the voltage‐clamp mode (Figure 1c), we also validated Kir4.1 specificity by using BaCl_2_. These recordings showed a significant reduction in I‐V current (Figure 1d) and in Kir4.1 current density (~1.6 fold) in *APOE3* (87.75 ± 38.98 pA/pF) compared to *APOE4* (55.38 ± 23.18 pA/pF) MCs, indicating compromised K^+^ buffering ability in *APOE4* MCs (*p* = 0.0001, Figure 1e).

### 3.2 Reduction in AQP4 in Retinas of *APOE4* Mice

Aquaporin (AQP4) is a key water channel enriched at the end feet of the MC, and it maintains water homeostasis. Given the interplay between K^+^ and water flux, particularly under gliotic or osmotic stress conditions, changes in AQP4 may reflect MC swelling and contribute to overall functional impairments (Nagelhus et al. 2004). Therefore, to determine whether *APOE4* causes MC swelling and affects osmotic regulation, we analyzed AQP4 expression in the retinal sections of *APOE3* and *APOE4* mice using immunofluorescence. AQP4 localized strongly to GCL and ILM, consistent with its distribution to MC end‐feet (Figure S1a). Quantitative analysis revealed a significant decrease in AQP4 fluorescence intensity in *APOE4* retinas compared to *APOE3* retinas (*p* = 0.0345, Figure S1b), indicating impaired water homeostasis. This suggests that *APOE4* may promote MC dysfunction by disturbing both K^+^ and water balance in the retina.

### 3.3 *APOE4* Retinas Show Elevated Cholesterol Accumulation Without Kir4.1 Colocalization

In order to ascertain the potential involvement of cholesterol, we first looked into transcriptomic data from our previously published studies (Abhyankar et al. 2025). APOE, Kcnj10, and some of the well‐known targets involved in cholesterol metabolism in the retina (Léger‐Charnay et al. 2022) were included for pathway analysis. These targets were then independently searched for available networks using the pathway ‘grow’ function (Figure S1). IPA predicted activation of 3‐hydroxy‐3‐methyl‐glutaryl‐coenzyme A reductase (HMGCR) in *APOE4*, consistent with upregulated cholesterol biosynthesis (Figure S2a). However, no transcriptional link between HMGCR and KCNJ10 (Kir4.1) was identified. To address potential membrane‐level regulation, we stained retinal sections from *APOE3* and *APOE4* mice with BODIPY (cholesterol/lipid marker) and Kir4.1. *APOE4* retinas showed increased BODIPY accumulation compared with *APOE3*, but no colocalization with Kir4.1 (Figure S2b).

### 3.4 *APOE4* Allele Leads to Mitochondrial Dysfunction

**FIGURE 2:**
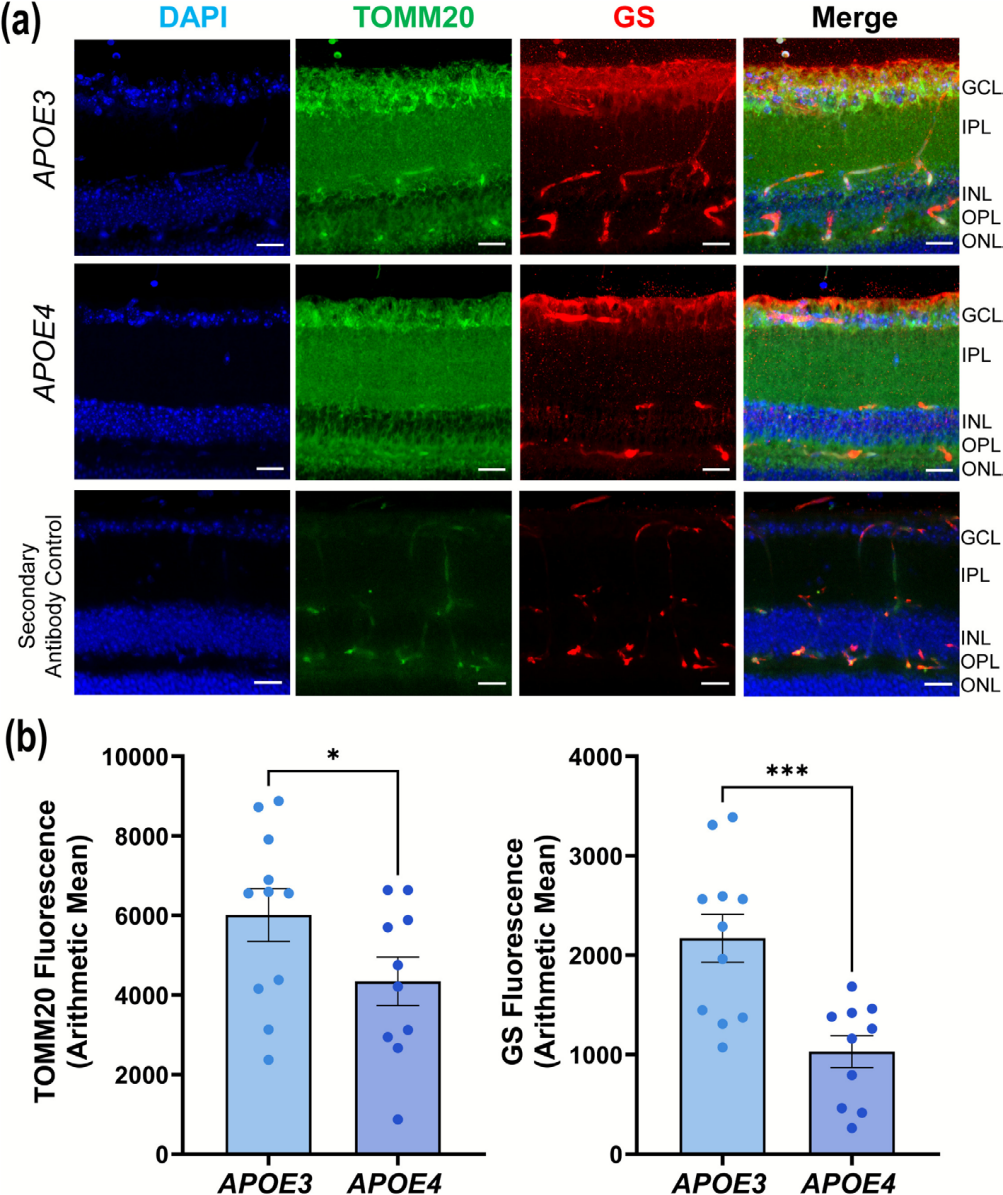
Mitochondrial dysfunction in *APOE4*. (a) Representative images of retinal slices showing glutamine synthase (GS) and TOMM20 staining pattern in *APOE3* and *APOE4* mice, scale 20 μm (*n*: *APOE3* = 3, *APOE4* = 3). (b) Bar graph showing quantification of immunofluorescence for TOMM20 and GS (*n*: 10–11 images/group). Values are expressed as mean ± SEM. An unpaired *t*‐test was used for statistical analysis. *p < 0.05, ***p < 0.001.

Mitochondrial impairment is well documented in AD (Wang et al. 2020). The *APOE4* allele has been shown to disrupt mitochondrial gene expression by acting as a transcriptional factor or by directly interacting with mitochondria, altering metabolism and fusion/fission balance, resulting in reduced ΔΨm, ROS generation, and subsequent mitochondrial dysfunction (Chang et al. 2005). To investigate whether *APOE4* also induces mitochondrial deficits in the retina, we stained retinal sections from *APOE3* and *APOE4* mice with TOMM20 (a mitochondrial marker) (Figure 2a). Retinas from *APOE4* mice displayed a marked reduction in TOMM20 fluorescence (*p* = 0.0404) along with GS (*p* = 0.0005) compared to *APOE3* retinas (Figure 2b), indicating a decrease in mitochondrial content or function. These findings suggest that the *APOE4* allele may contribute to the mitochondrial dysfunction in retinal cells, potentially linking broader cellular impairments seen in AD.

### 3.5 *APOE4* ‐Transfected rMC‐1 Have Lower Kir4.1 Gene and Protein Expression

**FIGURE 3:**
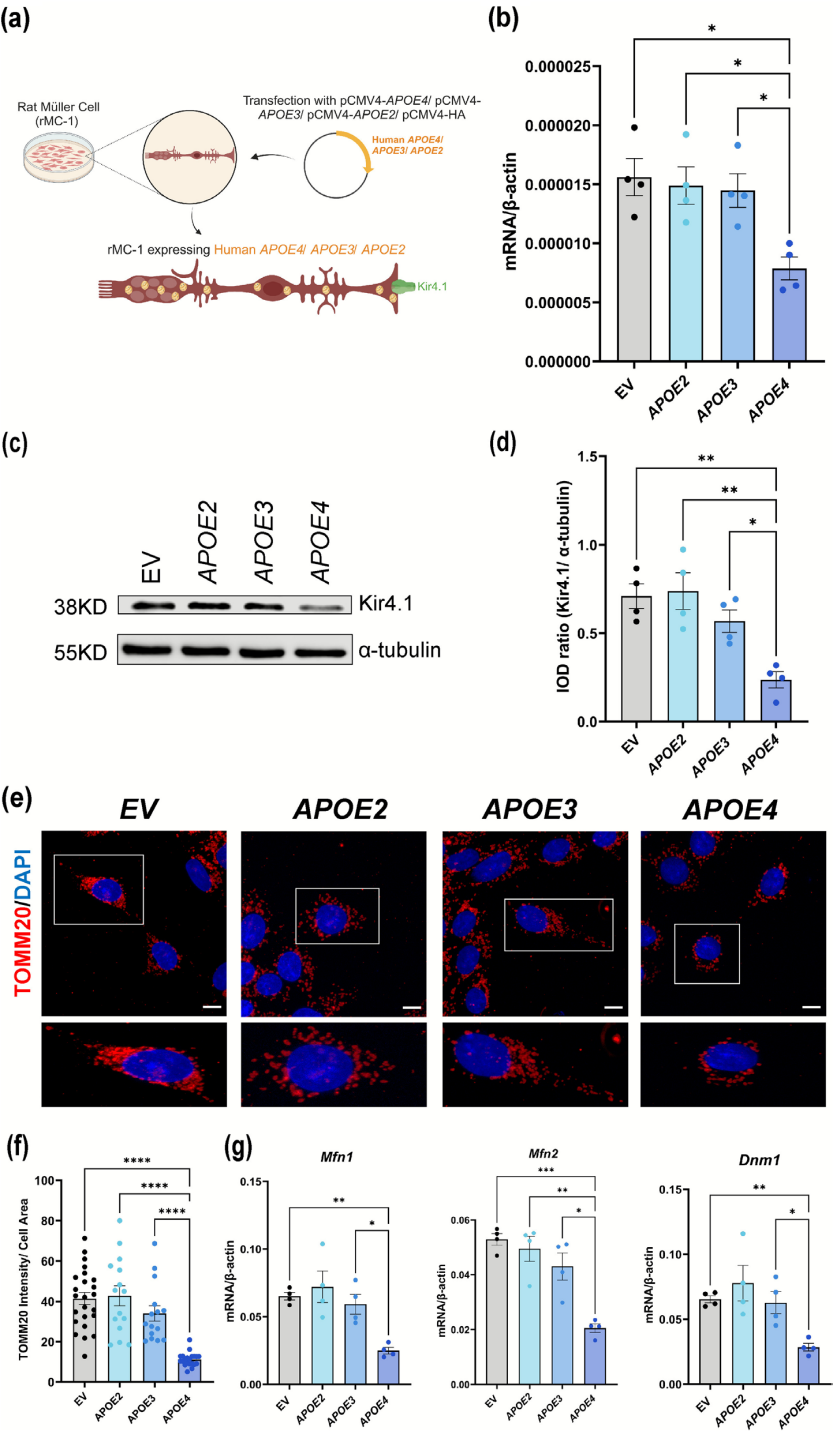
*APOE4* decreases Kir4.1 and mitochondrial expression in rMC‐1. (a) Schematic showing the generation of rMC‐1 expressing human *APOE* isoforms. rMC‐1 was transiently transfected with human *APOE2*/*APOE3*/*APOE4*, and EV was used as a control. (b) mRNA expression of *Kcnj10* gene for Kir4.1 normalized to a housekeeping gene β‐actin. (c) Representative western blots of Kir4.1 expression and (d) quantification of integrated optical density (IOD) ratio of Kir4.1 and α‐tubulin showing decreased protein expression of Kir4.1 in *APOE4*‐transfected rMC‐1. (e) Representative images of rMC‐1 transfected with human *APOE2*/*APOE3*/*APOE4*/EV showing decreased TOMM20 staining pattern in *APOE4*‐transfected rMC‐1, scale: 20 μm (*n*: 3 independent experiments). (f) Quantification of TOMM20 staining intensity per cell area (*n*: 15–24 cells/condition). (g) mRNA expression of *Mfn1*, *Mfn2*, and *Dnm1*, showing that *APOE4*‐transfected rMC‐1 reduced *Mfn1*, *Mfn2*, and *Dnm1* gene expression as compared to EV/*APOE2*/*APOE3*‐transfected rMC‐1 (*n*: 4 independent experiments). Values are expressed as mean ± SEM. One‐way ANOVA followed by Tukey's multiple comparison test was used for statistical analysis. *p < 0.05, **p < 0.01, ****p < 0.0001.

To further confirm our findings from the mouse model, we created an in vitro model by transfecting rMC‐1 with *APOE2*, *APOE3*, or *APOE4*, using an EV as a control (Figure 3a). While humans have three APOE variants, rats have only one, which contains arginine at 112 (https://web.expasy.org/variant_pages/VAR_000652.html), unlike human *APOE4*, and rat APOE is similar to human *APOE3*. First, we validated the transfection using immunofluorescence (Figure S3). The staining showed that rMC‐1 transfected with *APOE2*, *APOE3*, or *APOE4* plasmids exhibited distinct intracellular staining corresponding to the expressed APOE proteins, while the staining for anti‐HA showed transfection with EV, confirming the efficacy of the transfection and the expression of human APOE isoforms in rMC‐1. In line with the observations in retinal tissue, rMC‐1 transfected with *APOE4* showed a significant decrease in Kir4.1 gene expression (Figure 3b) compared to the cells transfected with EV (*p* = 0.0124) or *APOE2* (*p* = 0.0123) or *APOE3* (*p* = 0.0363). Western blot analysis supported these results, revealing a marked reduction in Kir4.1 protein levels in *APOE4*‐transfected rMC‐1 (Figure 3c) compared to the EV (*p* = 0.0032) or *APOE2* (*p* = 0.0020) or *APOE3* (*p* = 0.0340) (Figure 3d).

Since altered Kir4.1 expression in *APOE4* retinas was accompanied by increased lipid signal, we next examined whether a similar effect occurred in rMC‐1. Consistent with the in vivo results, *APOE4*‐transfected rMC‐1 displayed elevated BODIPY staining alongside reduced Kir4.1 expression, but again the two signals did not colocalize (Figure S4). These findings suggest that *APOE4* alters cholesterol handling and storage in MCs, while the reduction in Kir4.1 occurs through an independent mechanism.

### 3.6 Mitochondrial Deficits in *APOE4* ‐Transfected rMC‐1

We sought to assess mitochondrial health in *APOE4*‐transfected rMC‐1. We stained rMC‐1 transfected with EV/*APOE2*/*APOE3*/*APOE4* with TOMM20 (Figure 3e) and found that TOMM20 staining intensity (Figure 3e) is decreased in rMC‐1 transfected with *APOE4*, further confirming findings from in vivo staining. The TOMM20 staining intensity was found to be significantly decreased in *APOE4*‐transfected rMC‐1 compared to EV/*APOE2*/*APOE3* (*p* ≤ 0.0001) (Figure 3f). We examined mRNA expression of mitochondrial fusion genes *Mfn1* and *Mfn2* and fission gene *Dnm1* (Figure 3g). Results showed that *APOE4* transfection led to significant downregulation of *Mfn1*, *Mfn2*, and *Dnm1* expression compared to EV (*p* = 0.0097, 0.0002, 0.0022) or *APOE2* (*p* = 0.0749, 0.0078, 0.0924) or *APOE3* (*p* = 0.0313, 0.0253, 0.0398). These findings collectively reinforce the role of *APOE4* in mitochondrial dysfunction and Kir4.1 downregulation, suggesting a consistent mechanism of MC dysfunction both in vivo and in vitro.

### 3.7 *APOE4* Expression Impairs Mitochondrial Respiration and Alters Metabolic Responses in rMC‐1

**FIGURE 4:**
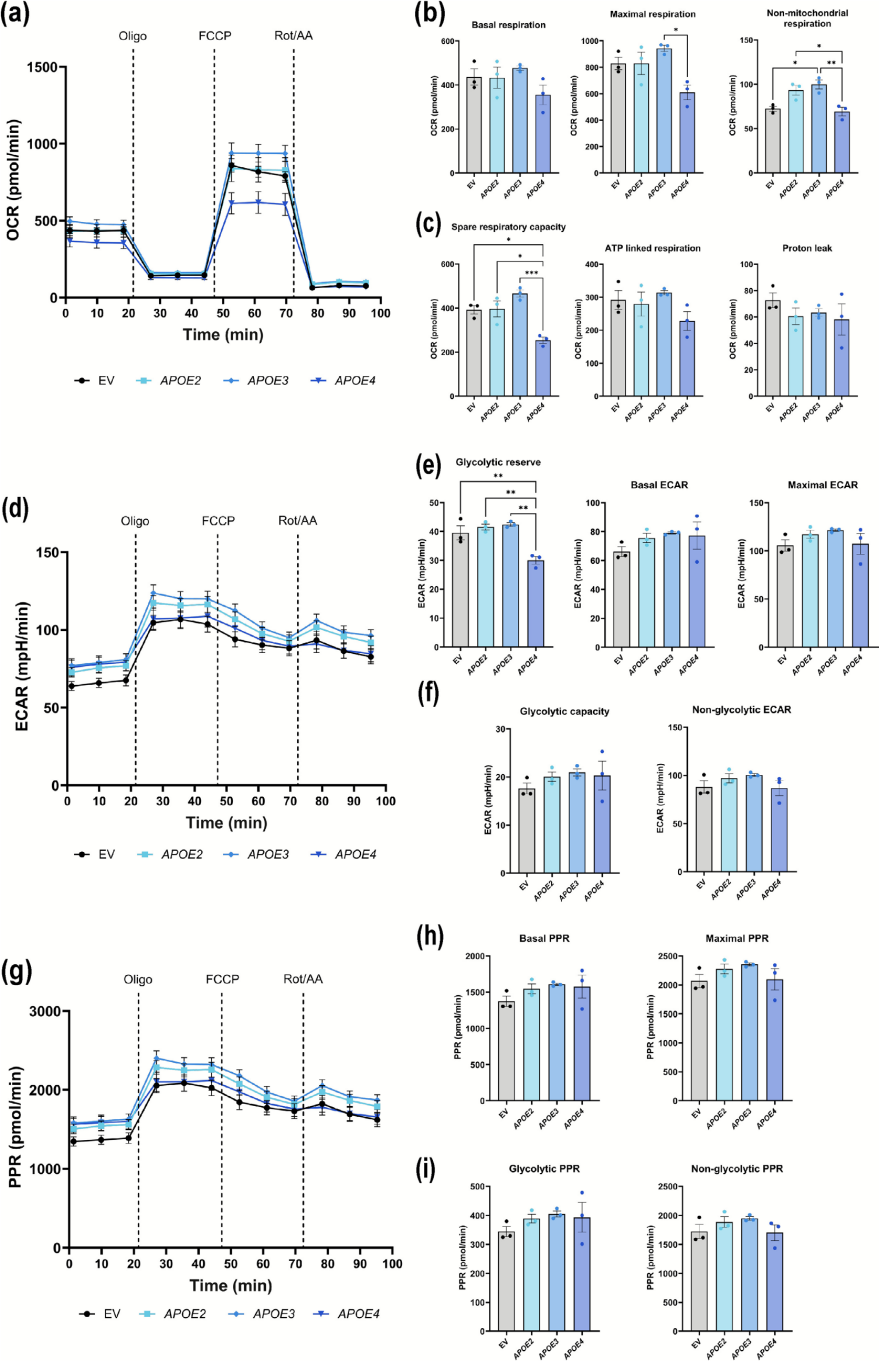
*APOE4* impairs mitochondrial respiration and reduces metabolic flexibility in rMC‐1. (a) OCR traces in rMC‐1 expressing EV/*APOE2*/*APOE3*/*APOE4* in response to sequential addition of oligomycin (oligo), FCCP, and rotenone/antimycin A (Rot/AA). *APOE4* expressing rMC‐1 showed consistently lower OCR across conditions. (b) Quantification of basal respiration, maximal respiration, and non‐mitochondrial respiration, with *APOE4* expressing rMC‐1 showing significantly reduced maximal and non‐mitochondrial respiration. (c) Quantification of spare respiratory capacity, ATP‐linked respiration, and proton leak. *APOE4*‐expressing rMC‐1 exhibited a marked reduction in spare respiratory capacity, while ATP‐linked respiration showed a downward trend. (d) ECAR profile in rMC‐1 expressing EV/*APOE2*/*APOE3*/*APOE4* in response to oligomycin (oligo), FCCP, and rotenone/antimycin A (Rot/AA) shows comparable basal rates across groups. (e) Quantification of glycolytic reserve, basal, and maximal ECAR. *APOE4* rMC‐1 displayed a significantly reduced glycolytic reserve compared to EV, *APOE2*, and *APOE3*‐transfected rMC‐1. (f) Quantification of glycolytic capacity and non‐glycolytic ECAR showing no significant changes across groups. (g) PPR traces in rMC‐1 expressing EV/*APOE2*/*APOE3*/*APOE4* in response to oligomycin (oligo), FCCP, and rotenone/antimycin A (Rot/AA) show overall comparable levels across groups. (h) Quantification of basal and maximal PPR confirms no significant APOE isoform differences. (i) Quantification of glycolytic PPR and non‐glycolytic PPR also showing no significant differences across groups (*n*: 3 independent experiments, with 3–4 technical replicates per condition). Values are expressed as mean ± SEM. One‐way ANOVA with Tukey's test was used for statistical analysis. *p < 0.05, **p < 0.01, ***p < 0.001.

MCs are highly metabolic and depend on both oxidative phosphorylation and glycolysis. *APOE4*'s impact on their metabolic flexibility is central to understanding retinal pathology. Therefore, we investigated APOE isoform‐specific effects on MC metabolism by using the Seahorse XF24 metabolic flux assay on rMC‐1 transfected with either EV/*APOE2*/*APOE3*/*APOE4*. Mitochondrial function was assessed by measuring the OCR over time. As shown in Figure 4a, *APOE4*‐transfected rMC‐1 consistently showed lower basal and maximal respiration compared to EV/APOE2/APOE3‐transfected rMC‐1. Though not statistically significant, *APOE4*‐transfected rMC‐1 exhibited the lowest basal respiration (Figure 4b), reflecting reduced baseline mitochondrial activity. Following the injection of FCCP, *APOE4*‐transfected rMC‐1 also showed a significant reduction in maximal respiration (Figure 4b, compared to *APOE3*, *p* = 0.0139), non‐mitochondrial respiration (Figure 4b, compared to *APOE2*, *p* = 0.0262, and *APOE3*, *p* = 0.0070), and spare respiratory capacity (Figure 4c, compared to EV, *p* = 0.0130; *APOE2*, *p* = 0.0110, and *APOE3*, *p* = 0.0009), indicating a diminished capacity to meet increased energy demands. Although not significant, APOE4‐transfected rMC‐1 showed lower ATP‐linked respiration (Figure 4c). Proton leak was comparable among EV and different APOE isoforms (Figure 4c). To assess glycolytic activity, the ECAR was measured. As shown in Figure 4d, all groups showed comparable ECAR following the injection of oligomycin (an ATP synthase inhibitor). *APOE4*‐transfected rMC‐1 showed significantly reduced glycolytic reserve (Figure 4e) as compared to EV (*p* = 0.0089), *APOE2* (*p* = 0.0028), or *APOE3* (*p* = 0.0018) transfected rMC‐1. No significant differences were observed for basal and maximal ECAR (Figure 4e) and glycolytic capacity and non‐glycolytic ECAR (Figure 4f). This suggests that APOE4‐transfected rMC‐1 cells have a greater reliance on glycolysis as a compensatory mechanism to produce ATP when mitochondrial respiration is inhibited. This elevated glycolytic activity likely serves to mitigate the energy deficits caused by the observed mitochondrial dysfunction. The PPR, a measure of total cellular energy output, was comparable between all the groups (Figure 4g). Basal and maximal PPR (Figure 4h) and glycolytic PPR and non‐glycolytic PPR (Figure 4i) were comparable between *APOE4*‐transfected rMC‐1 and other groups. Overall, these data show that *APOE4* is associated with significant mitochondrial dysfunction, characterized by lower respiration rates and increased proton leak. This metabolic impairment is partially compensated by an upregulation of glycolysis, but this compensatory mechanism appears insufficient to restore overall cellular energy balance, leading to a net bioenergetic deficit.

### 3.8 *APOE4* Impairs Mitochondrial Membrane Potential (ΔΨm) in rMC‐1

To assess ΔΨm in rMC‐1 expressing different *APOE* isoforms, we performed JC‐1 flow cytometry analysis. The results showed a notable decrease in ΔΨm in cells transfected with *APOE4* compared to those transfected with EV, *APOE2*, or *APOE3* (Figure S5a), suggesting that *APOE4* negatively impacts mitochondrial function. Quantitative analysis revealed a significant reduction in the ratio of red (aggregated JC‐1, indicating normal ΔΨm) to green (monomeric JC‐1, indicative of mitochondrial depolarization) fluorescence in *APOE4*‐expressing rMC‐1 compared to EV (*p* < 0.0001) or *APOE2* (*p* < 0.0001) or *APOE3* (*p* = 0.0001) (Figure S5b). This shift toward green fluorescence in *APOE4*‐transfected cells highlights a loss of mitochondrial membrane potential, a hallmark of mitochondrial dysfunction.

### 3.9 *APOE4* Increases Mitochondrial ROS Accumulation

To investigate oxidative stress within the mitochondria, we measured mitochondrial ROS levels in rMC‐1 cells transfected with *APOE4*, using MSR flow cytometry analysis (Figure S6a). *APOE4*‐expressing cells exhibited a significant increase in mitochondrial ROS production compared to cells expressing EV (*p* = 0.0003) or *APOE2* (*p* = 0.0032) or *APOE3* (*p* = 0.0282) (Figure S6b), indicating heightened oxidative stress, specifically associated with the *APOE4* isoform. This elevation in ROS further underscores the mitochondrial impairments linked to *APOE4*, contributing to cellular stress and potential damage within the retinal environment.

### 3.10 MitoQ Treatment Restores Kir4.1 Expression in *APOE4* ‐Transfected rMC‐1

To investigate whether mitochondrial‐targeted antioxidant MitoQ could mitigate mitochondrial dysfunction and restore Kir4.1 expression in *APOE4*‐transfected rMC‐1, we treated these cells with 1 μM MitoQ and assessed its impact on Kir4.1 levels. First, to evaluate the safety and potential toxicity of 1 μM MitoQ treatment on rMC‐1, we performed a viability assay using Alamar Blue in cells transfected with EV, *APOE2*, *APOE3*, and *APOE4* (Figure S7a). Following MitoQ treatment, we observed no reduction in cell viability compared to the vehicle‐treated controls across all the groups. Each group, including EV, *APOE2*, *APOE3*, and *APOE4*‐transfected cells, demonstrated ~100% viability after MitoQ exposure (Figure S7b), indicating that the treatment does not induce cytotoxic effects at the applied concentrations. This confirms that MitoQ is well‐tolerated by rMC‐1 cells and suitable for further experiments to improve mitochondrial function and cellular health in *APOE4*‐expressing cells.

**FIGURE 5:**
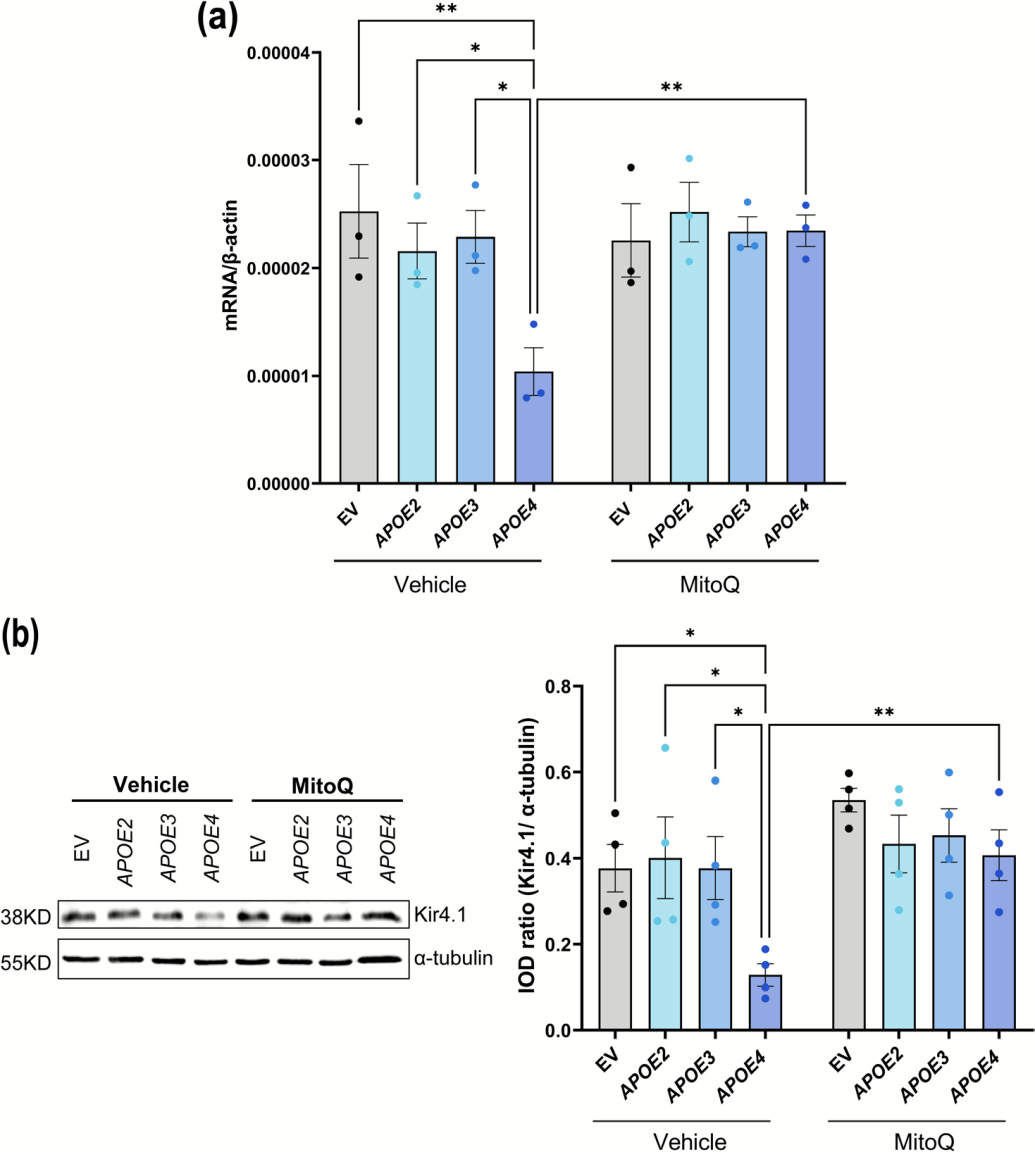
MitoQ restores Kir4.1 gene and protein expression in rMC‐1 transfected with *APOE4*. (a) mRNA expression of *Kcnj10* gene for Kir4.1 normalized to housekeeping gene for β‐actin after treating rMC‐1 with 1 μM MitoQ and vehicle. mRNA expression of Kir4.1 was significantly increased in *APOE4*‐transfected rMC‐1 upon treatment with 1 μM MitoQ compared to the vehicle. (b) Representative western blots of Kir4.1 expression and quantification of IOD ratio of Kir4.1 and α‐tubulin showing comparable protein expression of Kir4.1 in *APOE4*‐transfected rMC‐1 as compared to EV/*APOE2*/APOE3‐transfected rMC‐1 after treating with 1 μM MitoQ. Values are expressed as mean ± SEM. Two‐way ANOVA followed by Tukey's multiple comparison test was used for statistical analysis. *p < 0.05, **p < 0.01. (*n*: 3–4 independent experiments).

Since our main interest was Kir4.1 regulation, we next compared three different concentrations of MitoQ (0.5 μM, 1 μM, and 2 μM) for their effect on *Kcnj10* mRNA expression. Among these, 1 μM MitoQ significantly increased *Kcnj10* mRNA expression in *APOE4*‐transfected rMC‐1 compared to the 0.5 μM and 2 μM concentrations (Figure S8). Therefore, we performed the remaining experiments with 1 μM MitoQ. As seen earlier, following 1 μM MitoQ treatment, *APOE4*‐transfected cells significantly improved *Kcnj10* mRNA expression (*p* = 0.0037, Figure 5a) compared to vehicle‐treated *APOE4* cells. We observed that *APOE4*‐transfected cells have lower *Kcnj10* gene expression in vehicle‐treated EV (*p* = 0.0069) or *APOE2* (*p* = 0.0463) or *APOE3* (*p* = 0.0239). Similarly, *APOE4*‐transfected cells treated with MitoQ showed significantly improved Kir4.1 protein expression (*p* = 0.0041, Figure 5b) compared to vehicle‐treated *APOE4* cells. This increase in Kir4.1 expression in MitoQ‐treated *APOE4* cells brought them closer to levels observed in EV, *APOE2*, and *APOE3*‐transfected cells. These findings suggest that MitoQ, by enhancing mitochondrial health, can partially rescue Kir4.1 expression in *APOE4*‐expressing rMC‐1.

### 3.11 MitoQ Reduces Mitochondrial ROS in *APOE4* ‐Transfected rMC‐1 to Levels Comparable With *APOE2* /*APOE3‐*Transfected rMC‐1

**FIGURE 6:**
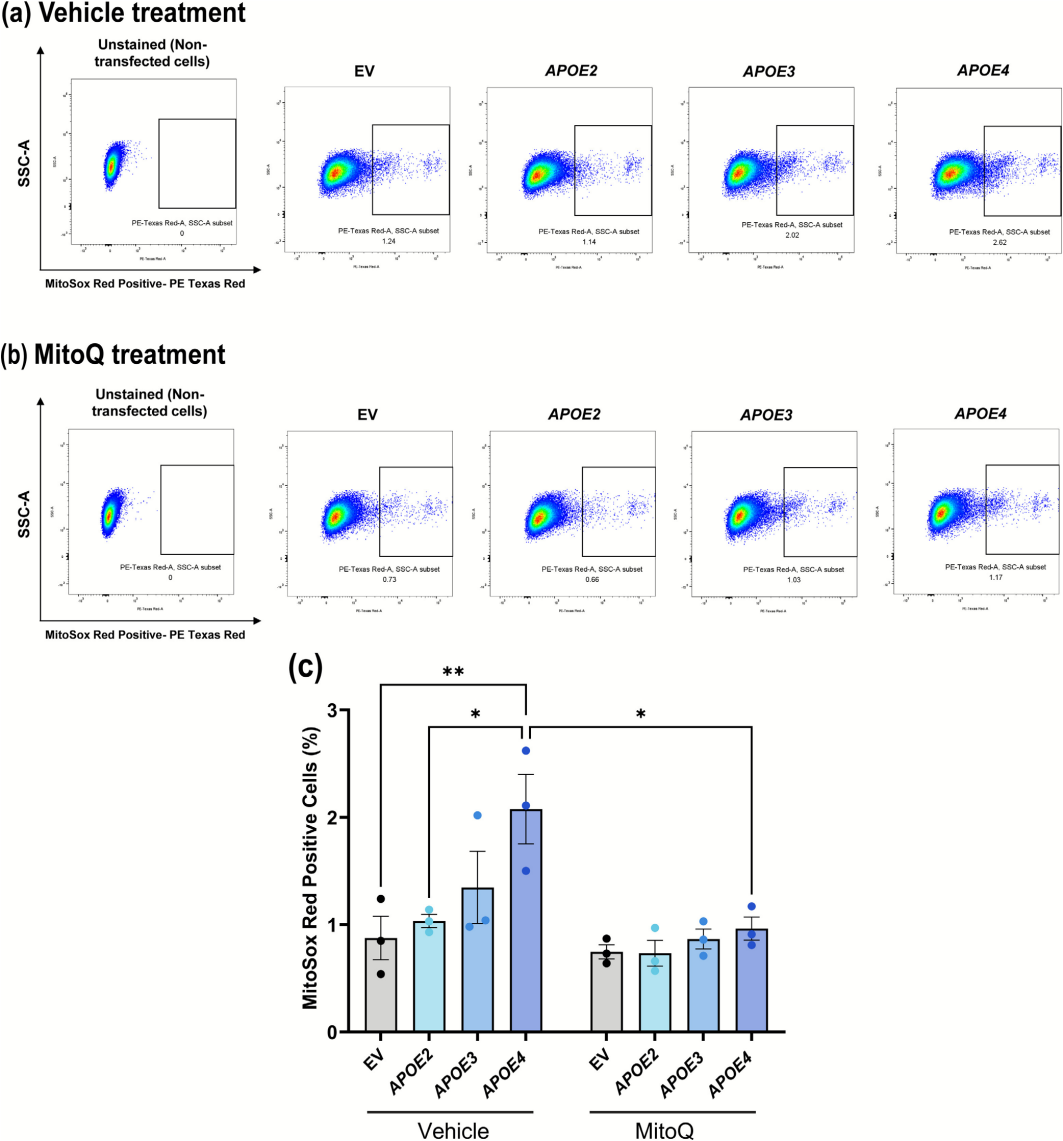
MitoQ decreases mitochondrial ROS in *APOE4*‐transfected rMC‐1. Representative images of unstained rMC‐1 and rMC‐1 transfected with EV/*APOE2*/*APOE3*/*APOE4* and treated with (a) vehicle or (b) MitoQ (1 μM). Cells were analyzed on a flow cytometer with 610/20 nm bandpass emission filter. (c) Bar graph showing quantification of % of MitoSox Red positive cells. Mitochondrial reactive oxygen species (ROS) was decreased upon treating *APOE4*‐transfected rMC‐1 with 1 μM MitoQ. Values are expressed as mean ± SEM (*n*: 3 independent experiments). One‐way ANOVA followed by Tukey's multiple comparison test was used for statistical analysis. *p < 0.05, **p < 0.01.

To further examine MitoQ's impact on mitochondrial oxidative stress in *APOE4*‐transfected cells, we conducted MSR flow cytometry to measure mitochondrial ROS levels following MitoQ treatment (Figure 6). Results indicated that MitoQ‐treated *APOE4*‐transfected rMC‐1 exhibited a significant reduction in mitochondrial ROS compared to vehicle‐treated *APOE4* rMC‐1 (*p* = 0.0162, Figure 6a,b). Also, vehicle‐treated *APOE4*‐transfected rMC‐1 had elevated ROS levels as compared to EV (*p* = 0.0088) or *APOE2* (*p* = 0.0266), and though not significant with *APOE3* (*p* = 0.206), there was a trend, suggesting *APOE3*‐transfected cells have lower mitochondrial ROS compared to *APOE4*‐transfected cells. Notably, this reduction in ROS by MitoQ treatment brought mitochondrial ROS levels in *APOE4* cells down to levels comparable with those observed in EV, *APOE2*, and *APOE3*‐transfected cells treated with either vehicle or MitoQ. These findings suggest that MitoQ effectively mitigates the elevated oxidative stress associated with the *APOE4* isoform, restoring mitochondrial ROS levels to those typical of *APOE2* and *APOE3* expression.

### 3.12 Casein Kinase (CK) Inhibition Increases Kir4.1 Expression in *APOE4* ‐Transfected rMC‐1

To identify potential regulatory pathways influencing Kir4.1 expression in the context of *APOE4*, we interrogated our retinal mRNA‐seq dataset (Abhyankar et al. 2025) using IPA. First, both APOE and KCNJ10 targets were included for pathway analysis. Further, these targets were independently searched for available networks using the pathway “grow” function. Overall, 588 nodes and 599 relationships were found in mammals when data from 1927 to December 2025 were queried. Next, we explored if there is a relationship between *APOE* and *Kcnj10*, and only 15 targets were connected with predicted relationships. These targets then overlapped with transcriptomic data from the above study, and the following four targets passed the threshold: APOE, *KCNJ10* (gene for Kir4.1), *CSNK2A1* (gene for casein kinase 2), *SORL1* (Sortilin‐Related Receptor 1) (Figure S9a). The CK was used for further experimentation, considering its regulatory role in a broad range of substrates and cellular processes. To further confirm the involvement of CK in regulating Kir4.1, we treated *APOE4*‐transfected rMC‐1 cells with selective CK1 and CK2 inhibitors and performed a western blot analysis (Figure S9b). In *APOE4*‐transfected cells, CK inhibition consistently elevated Kir4.1 and pushed expression up to or above baseline levels seen in EV+ vehicle‐treated cells. While not all changes reached statistical significance, Kir4.1 expression was significantly higher in *APOE2*+ CK1 compared with *APOE4*+ CK1 (*p* = 0.0237). In addition, EV+ vehicle cells showed significantly lower Kir4.1 compared with *APOE4*+ CK1 (*p* = 0.0245) and *APOE4*+ CK2 (*p* = 0.0345) (Figure S9b). The inhibition of CK led to an increase in Kir4.1 expression, supporting the findings of our retinal transcriptomic data. These data suggest that CK1/2 activity suppresses Kir4.1 expression and that inhibition can counteract *APOE4*‐driven deficits.

## 4 Discussion

Our study shows that the *APOE4* allele causes significant structural and functional deficits in MCs. These deficits are associated with mitochondrial content, disrupted gene expression, and increased ROS production in *APOE4*‐expressing MCs. Additionally, we emphasize that targeting mitochondrial impairments with antioxidants like MitoQ may offer a promising strategy for reducing the progression of retinal and neurodegenerative diseases. Our study used two complementary models to interrogate *APOE4*‐driven MC dysfunction: *APOE4*‐KI mice that chronically express the allele in vivo and rMC‐1 acutely transfected with human APOE isoforms. Despite differences in temporal context, both models converged on a shared mechanism characterized by reduced Kir4.1 expression and mitochondrial deficits. The *APOE4*‐KI mice reveal the cumulative consequences of lifelong APOE4 expression in a physiological setting, including sustained loss of MC homeostasis, retinal stress, and mitochondrial impairment. In contrast, the rMC‐1 model captures acute, cell‐autonomous effects of *APOE4*, demonstrating that *APOE4* alone is sufficient to impair Kir4.1 and mitochondrial metabolism. Together, these findings suggest that APOE4 acts through both chronic and immediate pathways to compromise MC function, providing a mechanistic link between early cellular changes and the broader neurodegenerative phenotype observed in vivo.

We observe a marked reduction in Kir4.1 channels and GS expression in *APOE4*‐*KI* retinas compared to *APOE3*‐*KI*, indicating that *APOE4* disrupts MC structural integrity. Functionally, these disruptions are compounded by a significant decrease in Kir4.1 channel current density, reflecting impaired K^+^ buffering capacity—a critical function of MCs. These findings align with previous studies showing reduced Kir4.1 mRNA levels in the medial temporal lobe of AD patients and with severe amyloid angiopathy, as well as reduced Kir4.1 mRNA and protein in APPSwDI/NOS2^−/−^ and APPSwDI mice (Wilcock et al. 2009). Conversely, upregulated Kir4.1 expression was found in the human AD cortex (Smith et al. 2022) and in the human AD middle temporal gyrus (Liu et al. 2024). Similarly, increased Kir4.1 mRNA and protein expression were observed in the dentate gyrus around amyloid plaques in APP/PS1 mice; however, K^+^ levels in the hippocampus and cortex remained unchanged (Huffels et al. 2022), suggesting that Kir4.1 function remained intact. Of note, our research is the first to show damage to the Kir4.1 structure and function in *APOE4*‐KI. Additionally, no previous studies have reported findings in human or mouse AD retinas. Kir4.1 does not function only as a homomeric channel; it can also form heteromers with Kir5.1, which alters its conductance and gating and changes how K+ is buffered (Hibino et al. 2004; Hibino et al. 2010; Ishii et al. 2003), and such complexes have been described in glia (Brasko et al. 2017), including MCs (Ishii et al. 2003). However, Kir5.1 function depends on Kir4.1, and in MCs, homomeric Kir4.1 is concentrated at the endfeet where K+ siphoning occurs, while Kir4.1/Kir5.1 heteromers are distributed in other compartments (Ishii et al. 2003). Thus, although heteromerization could provide partial compensation, reduced Kir4.1 in *APOE4* retinas would be expected to impair both homomeric and heteromeric channels, leaving MCs' K^+^ buffering insufficient.

Immunofluorescence staining showed a significant reduction in AQP4 in *APOE4*‐expressing retinas. Given AQP4's role in fluid transport and retinal integrity, its loss may promote MC swelling and increase neuroinflammation and metabolic stress. Mitochondrial health in astrocytic end‐feet is closely tied to perivascular channel expression; from the human brain samples of the idiopathic normal pressure hydrocephalus (dementia subtype), Hasan‐Olive et al. (2019) found pathological mitochondria at end‐feet strongly correlated with reduced perivascular AQP4 and increased astrogliosis (Hasan‐Olive et al. 2019). These data support a model in which mitochondrial dysfunction destabilizes AQP4, compromising perivascular homeostasis and glymphatic clearance. Our results, showing *APOE4*‐induced loss of both AQP4 and Kir4.1 alongside known mitochondrial deficits, point to a shared metabolic mechanism driving channel disruption, impaired neurovascular coupling, and reduced extracellular K^+^ clearance.

Because AQP4 and Kir4.1 are closely linked in MC endfeet, our next question was whether other membrane‐associated processes might also contribute to Kir4.1 disruption in *APOE4*. Cholesterol transport is central to membrane organization and ion channel stability (Beverley and Levitan 2024), raising the possibility that *APOE4* could influence Kir4.1 not only through transcriptional and signaling pathways, but also via lipid handling and membrane dynamics. However, although *APOE4* retinas showed increased BODIPY staining and transcriptomic activation of cholesterol synthesis genes (HMGCR), we found no spatial colocalization of BODIPY signal with Kir4.1 in retinal sections or rMC‐1 cells. While these findings point to altered lipid metabolism in *APOE4*, they did not show a clear relationship with Kir4.1 localization.

The link between *APOE4* and Kir4.1 dysfunction underscores the importance of glial cells in AD pathology, where glial dysfunction often precedes neuronal loss. Using an in vitro model of rMC‐1, we confirmed the *APOE4*‐specific downregulation of Kir4.1 at both the transcript and protein levels. The consistency between in vivo and in vitro findings reinforces the relevance of our model and highlights the specific impact of the *APOE4* isoform on MC dysfunction. Notably, *APOE2* and *APOE3* transfections did not replicate these deficits, further emphasizing the unique pathogenic role of *APOE4*.

Our findings highlight the role of mitochondria in *APOE4*‐mediated dysfunction, evidenced by reduced TOMM20 expression and mitochondrial content in *APOE4* retinas. It is worth mentioning that we observed a drastic reduction in TOMM20 expression levels from retinal sections and rMC‐1 in *APOE4* vs. *APOE3*. The in vitro system lacks systemic regulation and reflects more acute *APOE4* expression, potentially leading to a more pronounced mitochondrial phenotype. Additionally, rMC‐1, being immortalized, may have altered baseline mitochondrial dynamics, amplifying the effects of *APOE4*.

Human *APOE4* carriers show lower MFN1, MFN2, DNM1, and sirtuin‐3 in the brain (Yin et al. 2020), suggesting compromised mitochondrial biogenesis and function. We observed that *APOE4*‐transfected rMC‐1 exhibited significant reductions in the expression of mitochondrial fusion and fission genes (*Mfn1*, *Mfn2*, and *Dnm1*), indicating disrupted mitochondrial dynamics. In our study, *APOE4*‐transfected rMC‐1 exhibited marked metabolic impairment characterized by reduced OCR and diminished glycolytic reserve. These findings point to compromised oxidative phosphorylation without adequate glycolytic compensation, suggesting broad mitochondrial dysfunction accompanied by limited metabolic flexibility. These findings are consistent with results from primary astrocytes of *APOE4* mice, where OCR was reduced and glycolytic flux altered, suggesting a conserved glial phenotype of impaired bioenergetics adaptability (Farmer et al. 2021). Furthermore, these cells demonstrated impaired ΔΨm and increased mitochondrial ROS levels, hallmark features of mitochondrial dysfunction. These data are consistent with previous reports linking *APOE4* to disrupted mitochondrial biogenesis, oxidative stress, and deficits in ATP production (Liang et al. 2021; Orr et al. 2019; Troutwine et al. 2022) in N2a cells as well as brain tissues, critical contributors to neurodegenerative processes in AD. *APOE4* is associated with reduced mitochondrial antioxidant defenses, increased mitochondrial superoxide production, and oxidative damage to lipids and proteins (Marottoli et al. 2021). For AD patients carrying *APOE4*, elevated hydroxyl radicals in the blood (Ihara et al. 2000) and decreased cerebral oxygen consumption (Robb et al. 2022) have been observed, and neurons expressing *APOE4* demonstrate reduced ATP production (Orr et al. 2019). These findings underscore the profound impact of *APOE4* on mitochondrial dysfunction, highlighting its potential role in exacerbating oxidative stress and energy deficits that contribute to neurodegenerative processes in AD.

Our study provides promising evidence for the therapeutic potential of MitoQ, a mitochondrial‐targeted antioxidant, in mitigating *APOE4*‐induced MC dysfunction. MitoQ effectively reduced mitochondrial ROS levels in *APOE4*‐transfected cells, restoring them to levels observed in *APOE3* and *APOE2*‐transfected cells. Additionally, MitoQ treatment rescued Kir4.1 gene and protein expression in *APOE4* cells, bringing them closer to baseline levels seen in *APOE3*‐expressing cells. These results suggest that MitoQ alleviates oxidative stress and addresses the downstream consequences of mitochondrial dysfunction, thereby improving MC health and function.

Since we found that mitochondrial deficits impair Kir4.1 function, we next asked what upstream pathways might regulate its expression. Using our mRNA‐seq dataset and IPA, we identified both a direct regulatory link between *APOE4* and *KCNJ10*, as well as indirect regulation through CK signaling. CK has previously been implicated in ion channel modulation, and in our analysis, it emerged as an *APOE4*‐associated node predicted to influence Kir4.1 expression. This prompted us to test whether pharmacologic inhibition of CK1 and CK2 could restore Kir4.1 levels in MCs. Pharmacologic inhibition of CK1 and CK2 consistently increased Kir4.1 expression in *APOE4*‐expressing cells, suggesting that CK activity may act as a negative regulator of Kir4.1. These findings support our transcriptomic prediction that CK signaling contributes to *APOE4*‐mediated suppression of Kir4.1. Although the magnitude of rescue was modest and not uniformly significant across groups, the results provide mechanistic evidence linking *APOE4*, CK pathways, and Kir4.1 regulation. Targeting CK signaling may therefore represent a strategy to alleviate *APOE4*‐associated glial dysfunction.

The retinal findings in this study reflect broader pathological changes observed in the brain during AD, underscoring the retina's usefulness as a non‐invasive model for studying neurodegenerative diseases. Considering the role of MCs in maintaining the blood‐retinal barrier and supporting neuronal health, targeting mitochondrial dysfunction in these cells may offer a dual benefit of preserving retinal and brain health. The therapeutic effects of MitoQ observed here support its potential as a candidate for further clinical investigation settings (Young and Franklin 2019). Additionally, a long‐term and longitudinal study in which MitoQ is administered either systemically or locally would be helpful in reinforcing the protective role in AD in the mouse model; however, such a study was not feasible at this time. We acknowledge this as a limitation of our study, but our work paves the way for future research in this area. A study on 3xTg‐AD mice has shown that MitoQ inhibited cognitive decline in these mice (Young and Franklin 2019), and it was also shown to improve retinal function and reduce oxidative stress, inflammation, and apoptosis in a retinal ischemia–reperfusion injury rat model (Tang et al. 2022). Therefore, studying the effect of MitoQ, particularly on individuals carrying the *APOE4* allele who are at heightened risk for AD, might help improve their cognitive abilities.

While our findings offer crucial insights into *APOE4*‐induced MC dysfunction, several questions remain unanswered. For instance, the degree to which *APOE4*‐induced mitochondrial dysfunction directly drives other retinal pathologies, such as neuronal degeneration, requires further investigation. As discussed above, in vivo studies are essential to validate the therapeutic potential of MitoQ and establish optimal dosing regimens. Future research should also examine whether other mitochondrial‐targeted therapies or combination treatments could synergistically address *APOE4*‐associated retinal and neurodegenerative impairments.

In summary, our study identifies a novel mechanism by which *APOE4* impairs MC function through mitochondrial dysfunction, resulting in reduced Kir4.1 expression and K^+^ buffering capacity. MitoQ's ability to alleviate these deficits highlights the potential of targeting mitochondrial health as a therapeutic strategy for *APOE4*‐associated retinal and neurodegenerative diseases. These findings underscore the need to explore mitochondrial therapeutics in the context of *APOE4* AD.

## Author Contributions

S.D.A.: writing – original draft, writing – review and editing, conceptualization, software, validation, formal analysis, investigation. Y.X.: software, validation, formal analysis, investigation, writing – review and editing. N.M.: writing – review and editing. Q.L.: writing – review and editing, validation, formal analysis, investigation. T.R.C., A.L.O., and B.T.L.: resources, writing – review and editing. T.W.C.: conceptualization, writing – review and editing. A.D.B.: conceptualization, resources, writing – review and editing, supervision, project administration, funding acquisition.

## Funding

The authors acknowledge the funding support from the National Institute of Health (NIH)–National Eye Institute (NEI) grant R01EY027779‐S1, R01EY032080, and an Unrestricted grant from Research to Prevent Blindness (RPB) to A.D.B. S.D.A. was supported in part by the Indiana University Diabetes and Obesity Training Program (NIH T32), DK064466, and Sigma Xi Grants in Aid of Research (GIAR) G20240315‐8762.

## Conflicts of Interest

A.D.B. is an *ad ho* *c* District Support Pharmacist at CVS Health/Aetna. The contents of this study do not reflect those of CVS Health/Aetna. Y.X., N.M., Q.L., T.W.C., A.L.O., B.T.L., and S.D.A. do not have any conflicts to declare.

## Supporting information


**Figure S1:** glia70119‐sup‐0001‐FigureS1‐S9.docx. *APOE4* leads to a reduction in AQP4 expression in the retina. (a) Immunofluorescence staining images of retinal sections stained with AQP4 from 12 to 13 months old *APOE3* and *APOE4* mice. AQP4 is expressed in the ganglion cell layer (GCL) and inner limiting membrane (ILM), consistent with its distribution to MC end‐feet. The *APOE4* retinas showed a reduction in AQP4 expression compared to *APOE3* retinas, suggesting APOE4 potentially impacts retinal water homeostasis. Scale = 20 μm (*n*: *APOE3* = 3, *APOE4* = 3. Inner plexiform layer (IPL), outer plexiform layer (OPL), outer nuclear layer (ONL)). (b) Quantification of AQP4 fluorescence intensity shows significantly reduced expression in *APOE4* retinas (*n*: 11–12 images/group). Values are expressed as mean ± SEM. An unpaired *t*‐test was used for statistical analysis. **p < 0.01.
**Figure S2:** *APOE4* retinas show increased cholesterol accumulation independent of Kir4.1. (a) Ingenuity pathway analysis of retinal RNA‐seq data showing *APOE4*‐mediated activation of 3‐hydroxy‐3‐methyl‐glutaryl‐coenzyme A reductase (HMGCR), consistent with enhanced cholesterol biosynthesis. (b) Retinal agarose sections from *APOE3* and *APOE4* mice stained with Kir4.1 (green), and BODIPY (magenta). *APOE4* retinas show increased BODIPY signal (shown in white arrows) but no colocalization with Kir4.1. Scale= 20 μm. (*n*: 3 mice/group).
**Figure S3:** Confirmation of transfections. Representative images of rMC‐1 showing validation of transfection. rMC‐1 transfected with EV or human *APOE2*/*APOE3*/*APOE4* were stained for each antibody: anti‐HA (for EV), total APOE, APOE3 and APOE4. Scale 20 μm (*n*: 3 independent experiments).
**Figure S4:** *APOE4* expression in rMC‐1 increases cholesterol while reducing Kir4.1. rMC‐1 transfected with EV/*APOE2/APOE3/APOE4* and stained for Kir4.1 (green), and BODIPY (magenta). *APOE4*‐transfected cells show elevated BODIPY and reduced Kir4.1 expression, without colocalization between the two signals. Scale= 20 μm (*n*: 3 independent experiments).
**Figure S5:** *APOE4* decreases Mitochondrial membrane potential (ΔΨm) in rMC‐1. (a) Representative images of unstained rMC‐1 and rMC‐1 transfected with EV/*APOE2*/*APOE3*/*APOE4* and analyzed on a flow cytometer with 525/50 nm and 582/15 nm bandpass emission filters. (b) Bar graph showing quantification of % of the cells positive for JC‐1 monomers. Values are expressed as mean ± SEM (*n*: 5 independent experiments). One‐way ANOVA followed by Tukey's multiple comparison test was used for statistical analysis. ***p < 0.001, ****p < 0.0001.
**Figure S6:** *APOE4* increases Mitochondrial ROS production in rMC‐1. (a) Representative images of unstained rMC‐1 and rMC‐1 transfected with EV/*APOE2*/*APOE3*/*APOE4* and analyzed on a flow cytometer with 610/20 nm bandpass emission filter. (b) Bar graph showing quantification of % of MitoSox Red positive cells. Values are expressed as mean ± SEM (*n*: 4 independent experiments). One‐way ANOVA followed by Tukey's multiple comparison test was used for statistical analysis. *p < 0.05, **p < 0.01, ***p < 0.001.
**Figure S7:** MitoQ does not affect cell viability in rMC‐1. (a) Representative images of Alamar Blue‐treated rMC‐1 transfected with EV/*APOE2*/*APOE3*/*APOE4*. Untreated cells and 20% DMSO‐treated cells were used as control. (b) Bar graph showing quantification of % of cell viability, showing that 1 μM MitoQ treated rMC‐1 are viable compared to vehicle. Values are expressed as mean ± SEM (*n*: 3 independent experiments).
**Figure S8:** MitoQ (1 μM) is the optimal dose to restore Kir4.1 gene expression in *APOE4‐*transfected rMC‐1. mRNA expression of *Kcnj10* gene for Kir4.1 normalized to housekeeping gene β‐actin after treating rMC‐1 with three different doses of MitoQ: 0.5 μM, 1 μM and 2 μM and vehicle. mRNA expression of Kir4.1 was significantly increased in *APOE4*‐transfected rMC‐1 upon treatment with 1 μM MitoQ compared to the vehicle. Values are expressed as mean ± SEM. One‐way ANOVA followed by Tukey's multiple comparison test was used for statistical analysis. *p < 0.05, **p < 0.01 (*n*: 3 independent experiments).
**Figure S9:** Casein kinase (CK) inhibition increases Kir4.1 expression in rMC‐1 transfected with *APOE4*. (a) Ingenuity Pathway Analysis (IPA) of mRNA‐seq data from the retinas of *APOE3* and *APOE4* mice, highlighting a relationship between *APOE* and *Kcnj10*. Only 15 targets related to predicted relationships in between *APOE* and *Kcnj10*, and the following four targets passed the threshold: APOE, *KCNJ10* (gene for Kir4.1), *CSNK2A1* (gene for casein kinase 2), *SORL1* (Sortilin‐Related Receptor 1). *CSNK2A1* (circled in green) showed involvement in regulating Kir4.1. (b) Representative western blots showing Kir4.1 and α‐tubulin expression in rMC‐1 transfected with EV/*APOE2/APOE3/APOE4* and treated with CK1 inhibitor (CK1) or CK2 inhibitor (CK2), or vehicle (V). Quantification of Kir4.1 normalized to α‐tubulin across conditions. CK1 and CK2 treatment increased Kir4.1 expression in all groups, with *APOE4*+ CK1 showing significantly higher Kir4.1 than *APOE2*+ CK1 (*p = 0.0237). EV+ vehicle was significantly lower than *APOE4*+ CK1 (*p = 0.0245) and *APOE4*+ CK2 (*p = 0.0345). Data are expressed as mean ± SEM. One‐way ANOVA followed by Tukey's multiple comparison test was used for statistical analysis. *p = 0.05. (*n*: 3 independent experiments).

## Data Availability

The data that support the findings of this study are available from the corresponding author upon reasonable request.
