# Supplementary material for: Müller Glial Kir4.1 Channel Dysfunction in APOE4‐KI Model of Alzheimer's Disease
Source: Glia. 2026 Jan 8;74(3):e70119. doi: 10.1002/glia.70119 (PMC12780660; doi:10.1002/glia.70119)

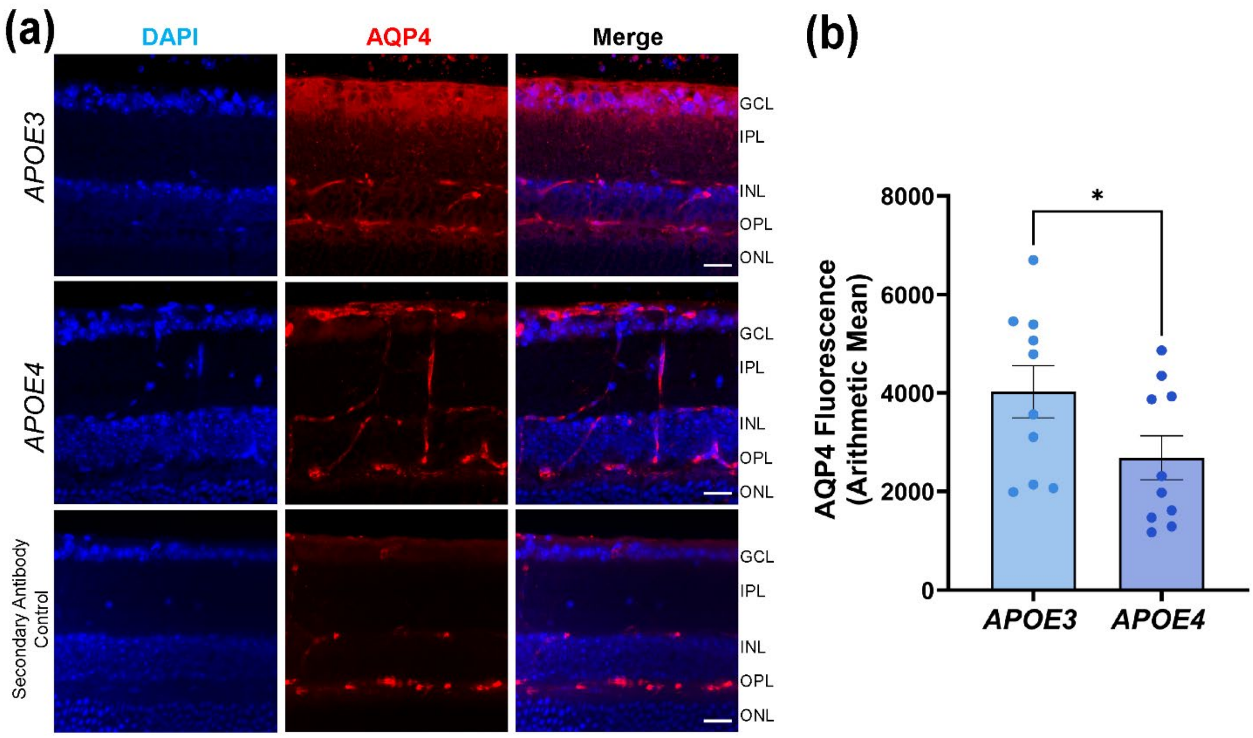

Figure S1: *APOE4* leads to a reduction in AQP4 expression in the retina. (a) Immunofluorescence staining images of retinal sections stained with AQP4 from 12-13 months old *APOE3* and *APOE4* mice. AQP4 is expressed in the ganglion cell layer (GCL) and inner limiting membrane (ILM), consistent with its distribution to MC end-feet. The *APOE4* retinas showed a reduction in AQP4 expression compared to *APOE3* retinas, suggesting APOE4 potentially impacts retinal water homeostasis. Scale= 20μm (n: *APOE3*= 3, *APOE4*= 3. Inner plexiform layer (IPL), outer plexiform layer (OPL), outer nuclear layer (ONL)). (b) Quantification of AQP4 fluorescence intensity shows significantly reduced expression in *APOE4* retinas. (n: 11-12 images/ group). Values are expressed as mean ± SEM. An unpaired t-test was used for statistical analysis. **p<0.01.

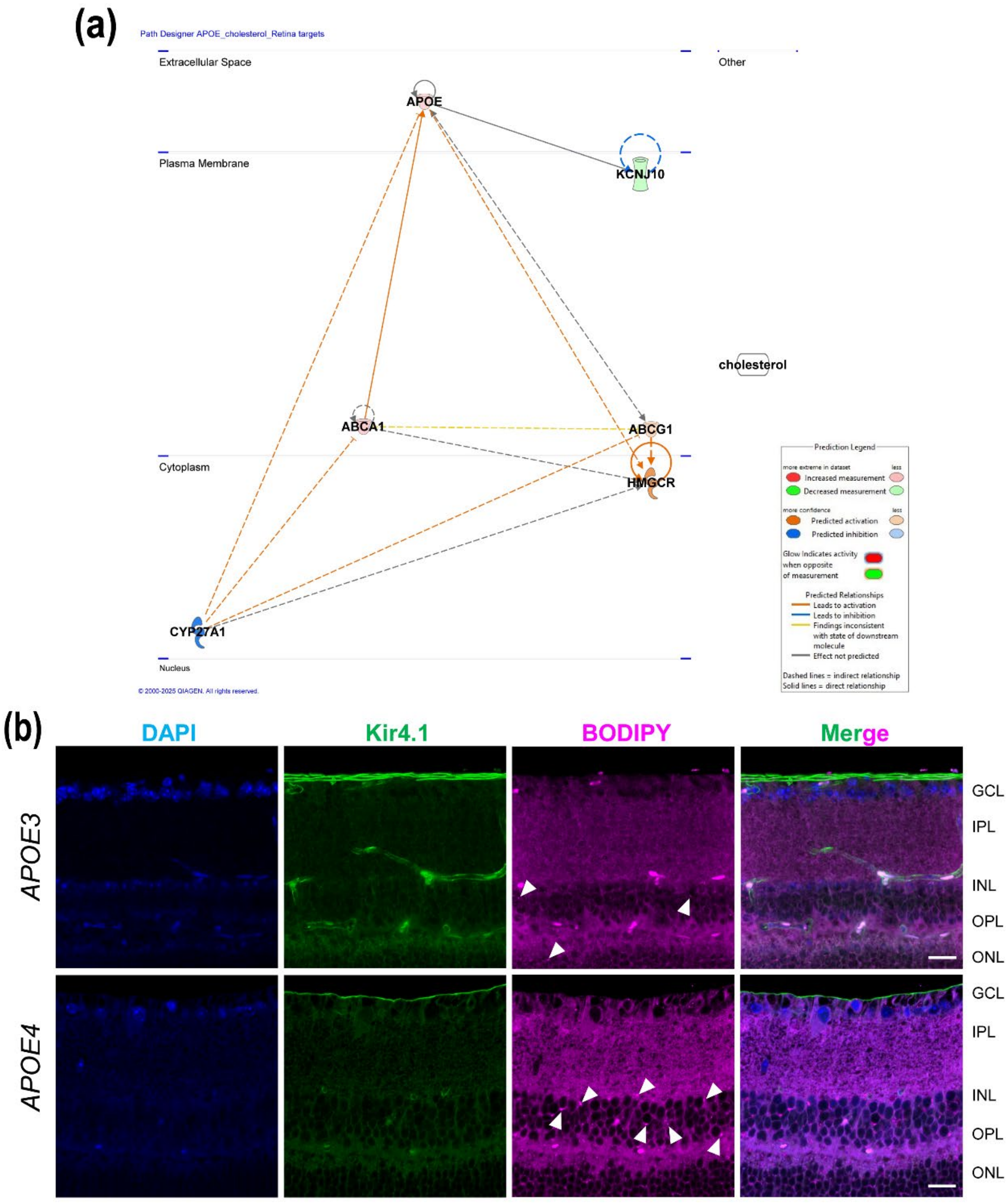

Figure S2: *APOE4* retinas show increased cholesterol accumulation independent of Kir4.1. (a) Ingenuity pathway analysis of retinal RNA-seq data showing *APOE4*-mediated activation of 3-hydroxy-3-methyl-glutaryl-coenzyme A reductase (HMGCR), consistent with enhanced cholesterol biosynthesis. (b) Retinal agarose sections from *APOE3* and *APOE4* mice stained with Kir4.1 (green), and BODIPY (magenta). *APOE4* retinas show increased BODIPY signal (shown in white arrows) but no colocalization with Kir4.1. Scale 20μm. (n: 3 mice/ group).

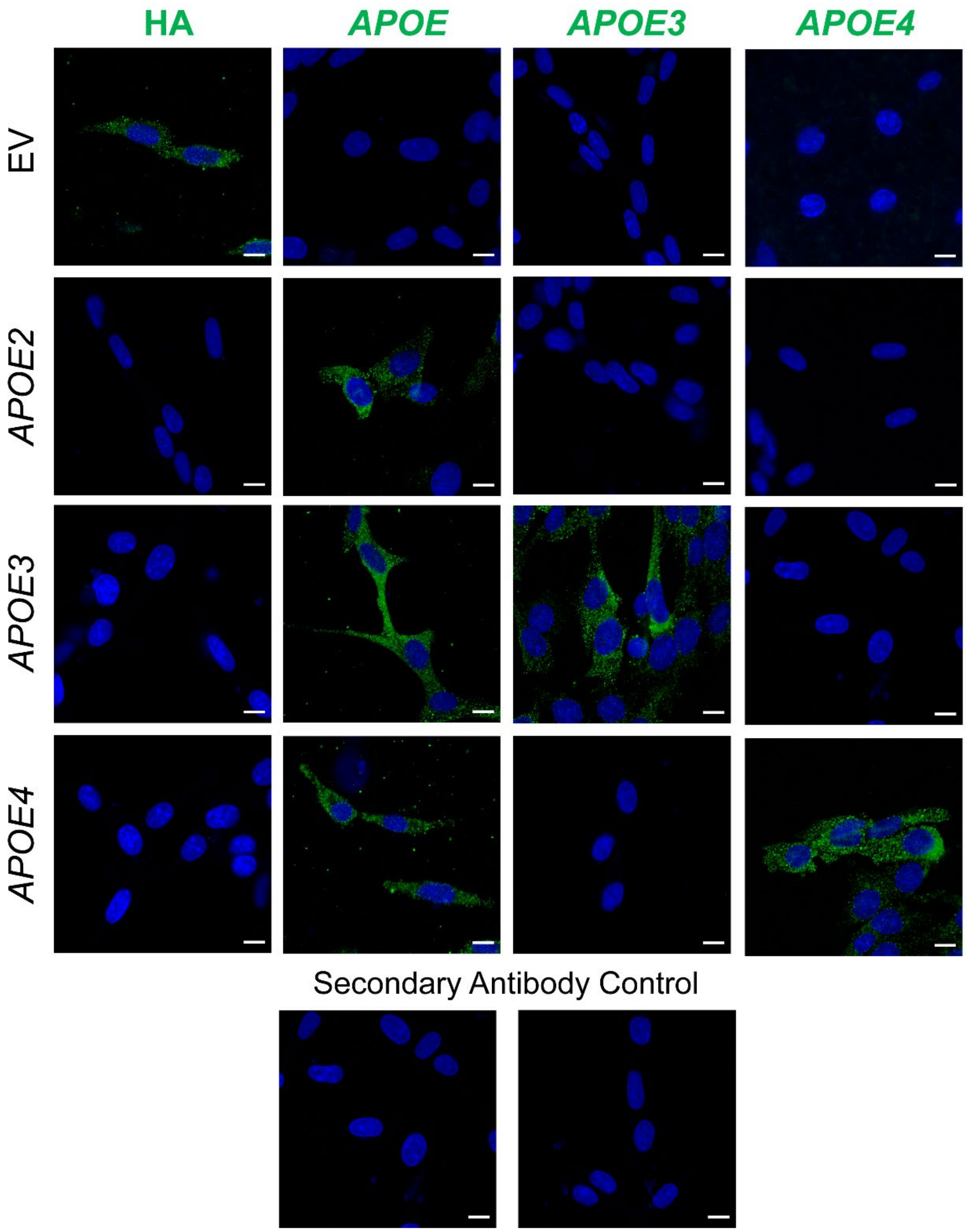

Figure S3: Confirmation of transfections. Representative images of rMC-1 showing validation of transfection. rMC-1 transfected with EV or human *APOE2*/ *APOE3*/ *APOE4* were stained for each antibody: anti-HA (for EV), total APOE, APOE3 and APOE4. Scale 20μm. (n: 3 independent experiments).

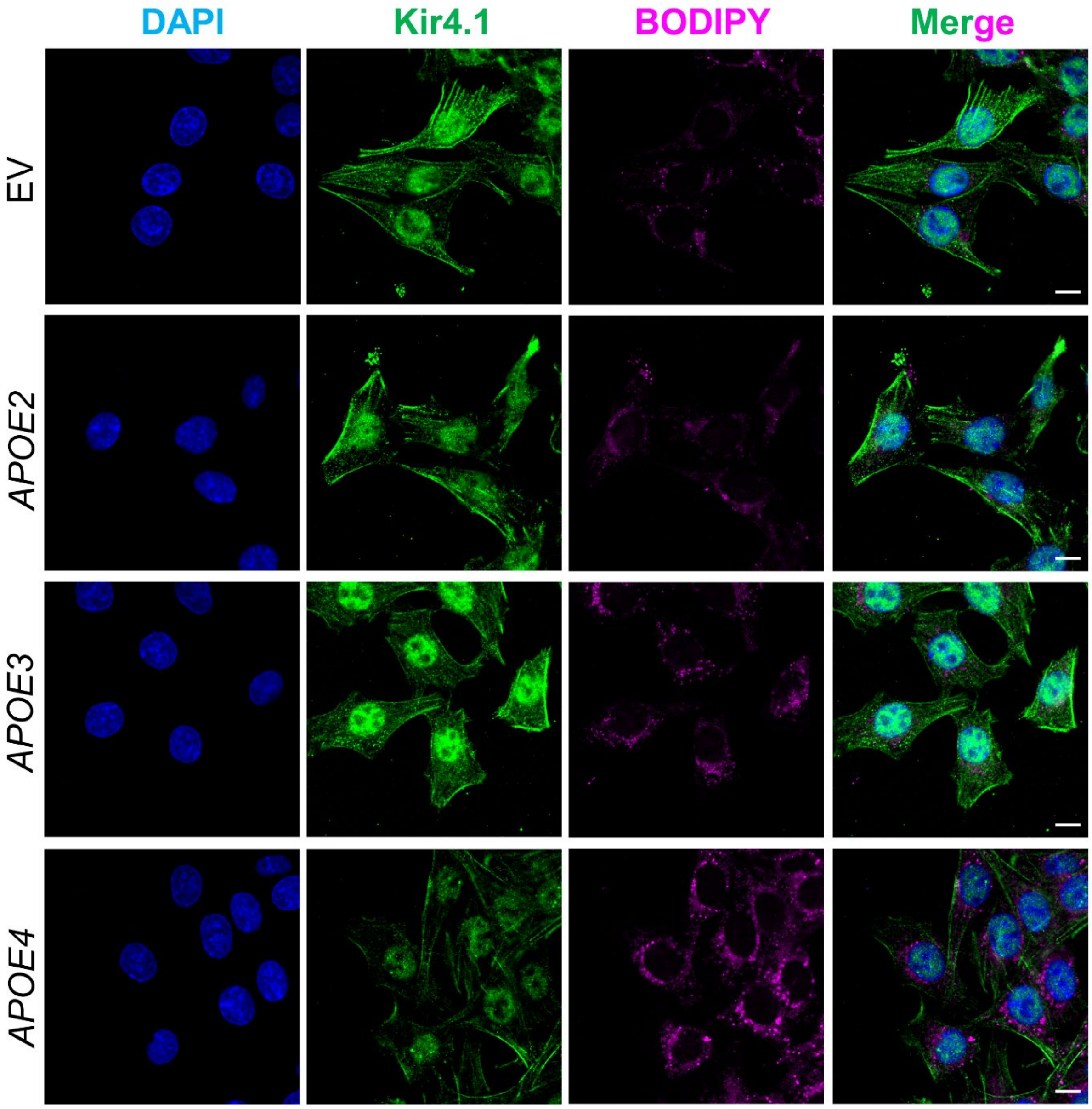

Figure S4: ***APOE4*** **expression in rMC-1 increases cholesterol while reducing Kir4.1.** rMC-1 transfected with EV/ *APOE2/ APOE3/ APOE4* and stained for Kir4.1 (green), and BODIPY (magenta). *APOE4*-transfected cells show elevated BODIPY and reduced Kir4.1 expression, without colocalization between the two signals. Scale bars: 20 µm. (n: 3 independent experiments).

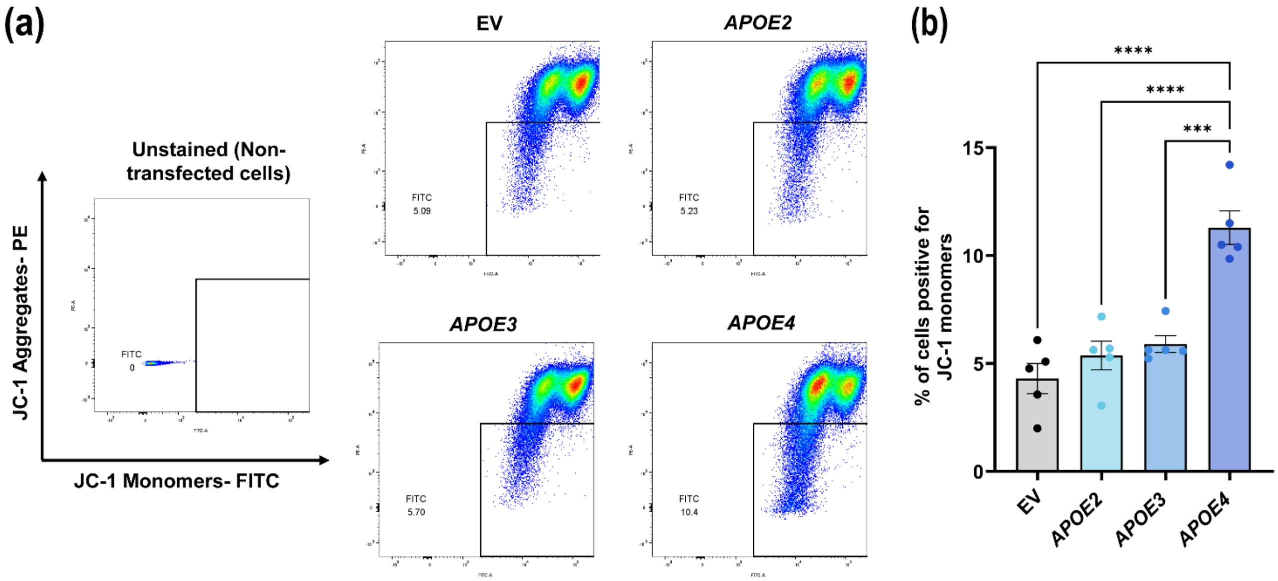

Figure S5: *APOE4* decreases Mitochondrial membrane potential (ΔΨm) in rMC-1. (a) Representative images of unstained rMC-1 and rMC-1 transfected with EV/ *APOE2*/ *APOE3*/ *APOE4* and analyzed on a flow cytometer with 525/50nm and 582/15nm bandpass emission filters. (b) Bar graph showing quantification of % of the cells positive for JC-1 monomers. Values are expressed as mean ± SEM (n: 5 independent experiments). One-way ANOVA followed by Tukey's multiple comparison test was used for statistical analysis. ***p<0.001, ****p<0.0001.

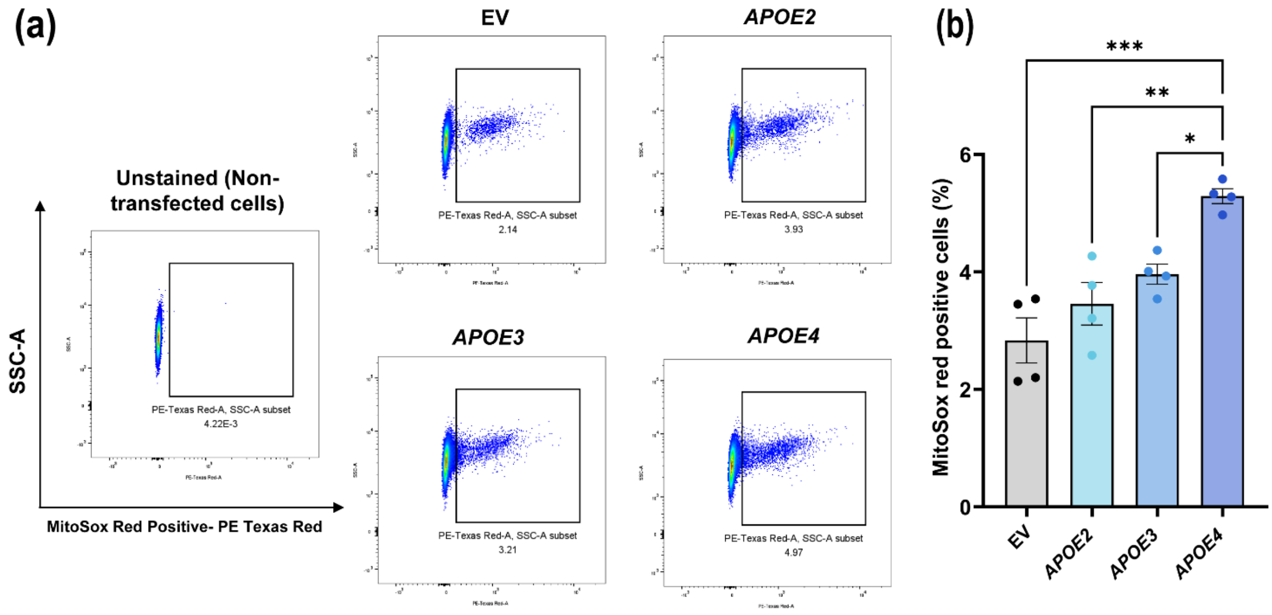

Figure S6: *APOE4* increases Mitochondrial ROS production in rMC-1. (a) Representative images of unstained rMC-1 and rMC-1 transfected with EV/*APOE2*/ *APOE3*/ *APOE4* and analyzed on a flow cytometer with 610/20nm bandpass emission filter. (b) Bar graph showing quantification of % of MitoSox Red positive cells. Values are expressed as mean ± SEM (n: 4 independent experiments). One-way ANOVA followed by Tukey's multiple comparison test was used for statistical analysis. $^*p<0.05$, $^{**}p<0.01$, $^{***}p<0.001$.

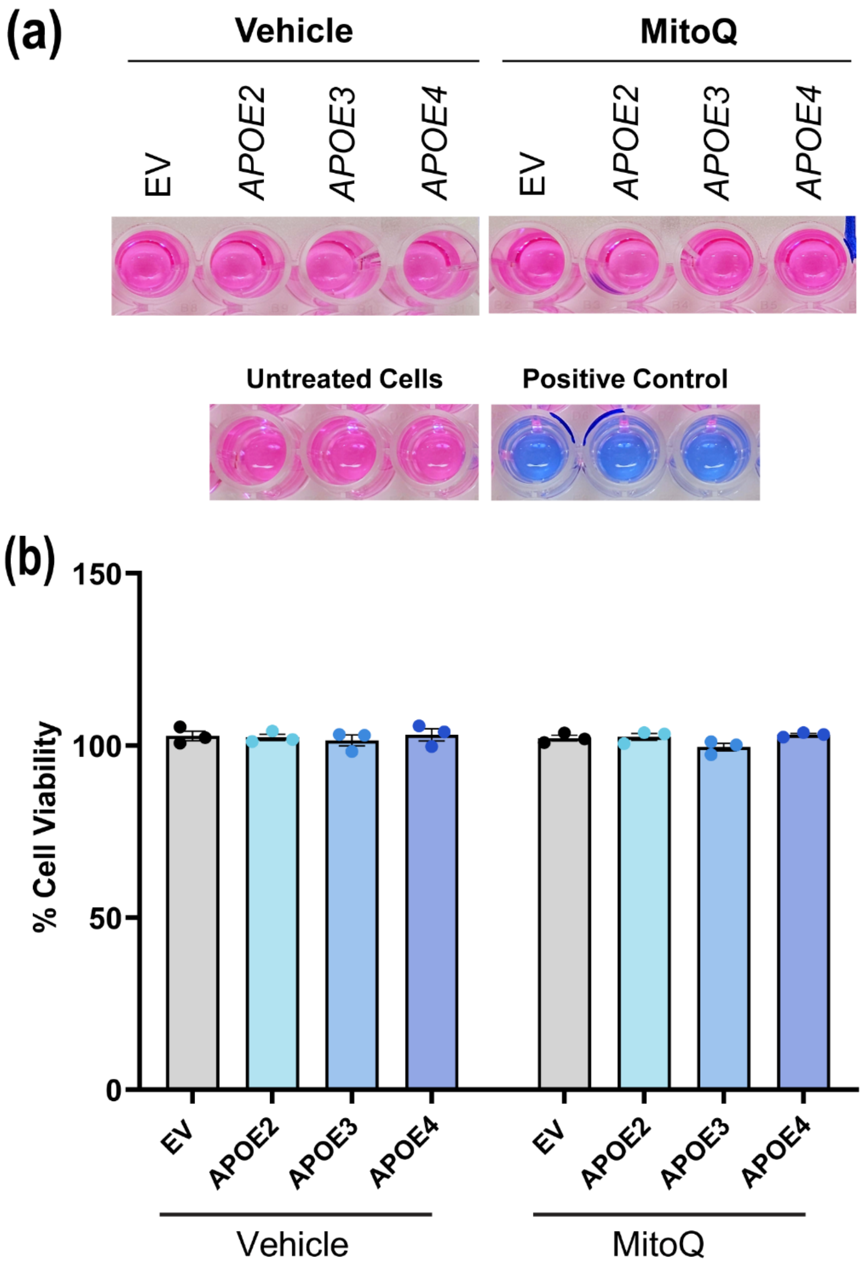

Figure S7: MitoQ does not affect cell viability in rMC-1. (a) Representative images of Alamar Blue-treated rMC-1 transfected with EV/ *APOE2*/ *APOE3*/ *APOE4*. Untreated cells and 20% DMSO-treated cells were used as control. (b) Bar graph showing quantification of % of cell viability, showing that 1µM MitoQ treated rMC-1 are viable compared to vehicle. Values are expressed as mean ± SEM (n: 3 independent experiments).

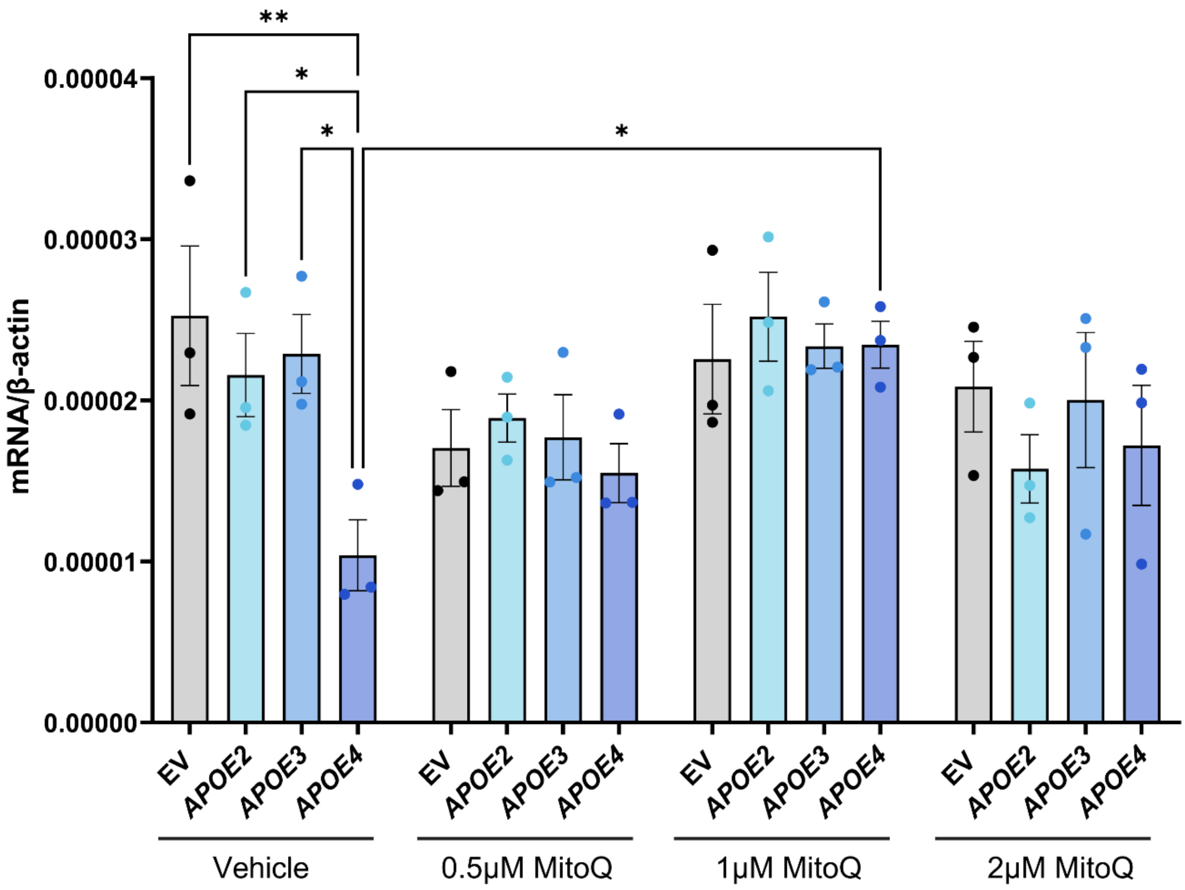

Figure S8: MitoQ (1μM) is the optimal dose to restore Kir4.1 gene expression in *APOE4*-transfected rMC-1. mRNA expression of *Kcnj10* gene for Kir4.1 normalized to housekeeping gene β-actin after treating rMC-1 with three different doses of MitoQ: 0.5 μM, 1μM and 2μM and vehicle. mRNA expression of Kir4.1 was significantly increased in *APOE4*-transfected rMC-1 upon treatment with 1μM MitoQ compared to the vehicle. Values are expressed as mean ± SEM. One-way ANOVA followed by Tukey's multiple comparison test was used for statistical analysis. *$p<0.05$, **$p<0.01$. (n: 3 independent experiments)

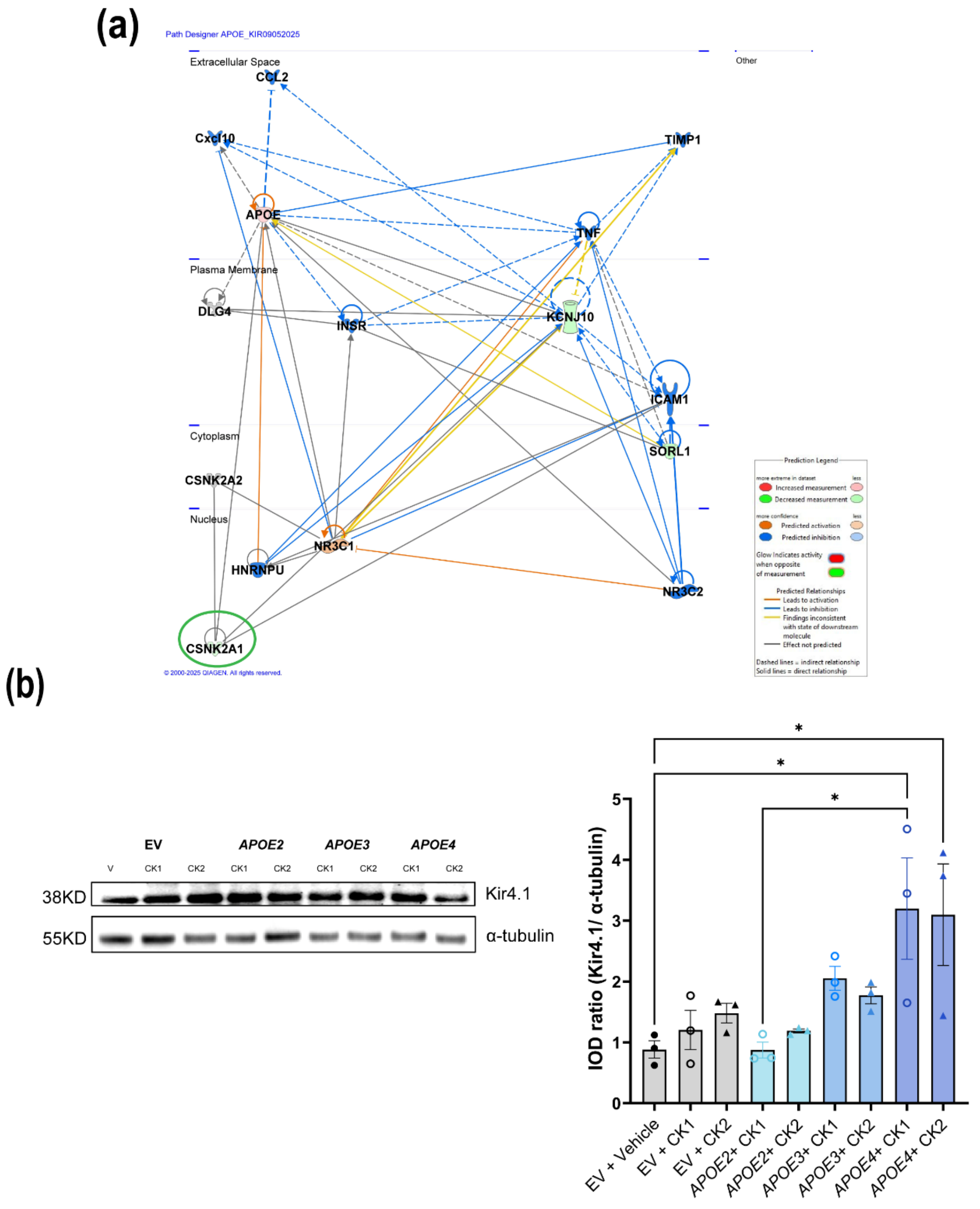

Figure S9: Casein kinase (CK) inhibition increases Kir4.1 expression in rMC-1 transfected with *APOE4*. (a) Ingenuity Pathway Analysis (IPA) from mRNA-seq data from the retinas of *APOE3* and *APOE4* mice, highlighting a relationship between *APOE* and *Kcnj10.* Only 15 targets related to predicted relationships in between *APOE* and *Kcnj10*, and the following four targets passed the threshold: APOE, *KCNJ10* (gene for Kir4.1), *CSNK2A1* (gene for casein kinase 2), *SORL1* (Sortilin-Related Receptor 1). *CSNK2A1* (circled in green) showed involvement in regulating Kir4.1. (b)

Representative western blots showing Kir4.1 and α-tubulin expression in rMC-1 transfected with EV/ *APOE2/ APOE3/ APOE4* and treated with CK1 inhibitor (CK1) or CK2 inhibitor (CK2), or vehicle (V). Quantification of Kir4.1 normalized to α-tubulin across conditions. CK1 and CK2 treatment increased Kir4.1 expression in all groups, with *APOE4*+ CK1 showing significantly higher Kir4.1 than *APOE2*+ CK1 (*p=0.0237). EV+ vehicle was significantly lower than *APOE4*+ CK1 (*p=0.0245) and *APOE4*+ CK2 (*p=0.0345). Data are expressed as mean ± SEM. One-way ANOVA followed by Tukey's multiple comparison test was used for statistical analysis. * p= 0.05. (n: 3 independent experiments).

Western blots for Figure 3

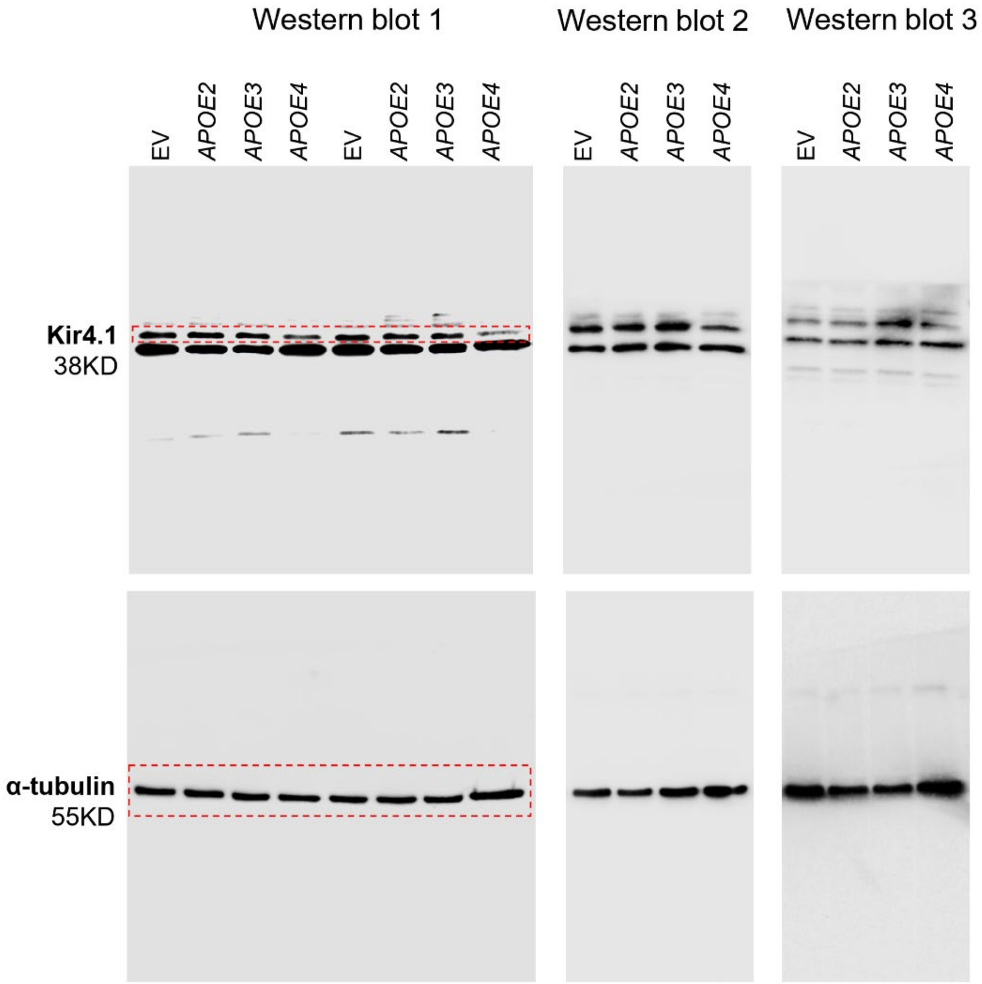

Western blots for Figure 5

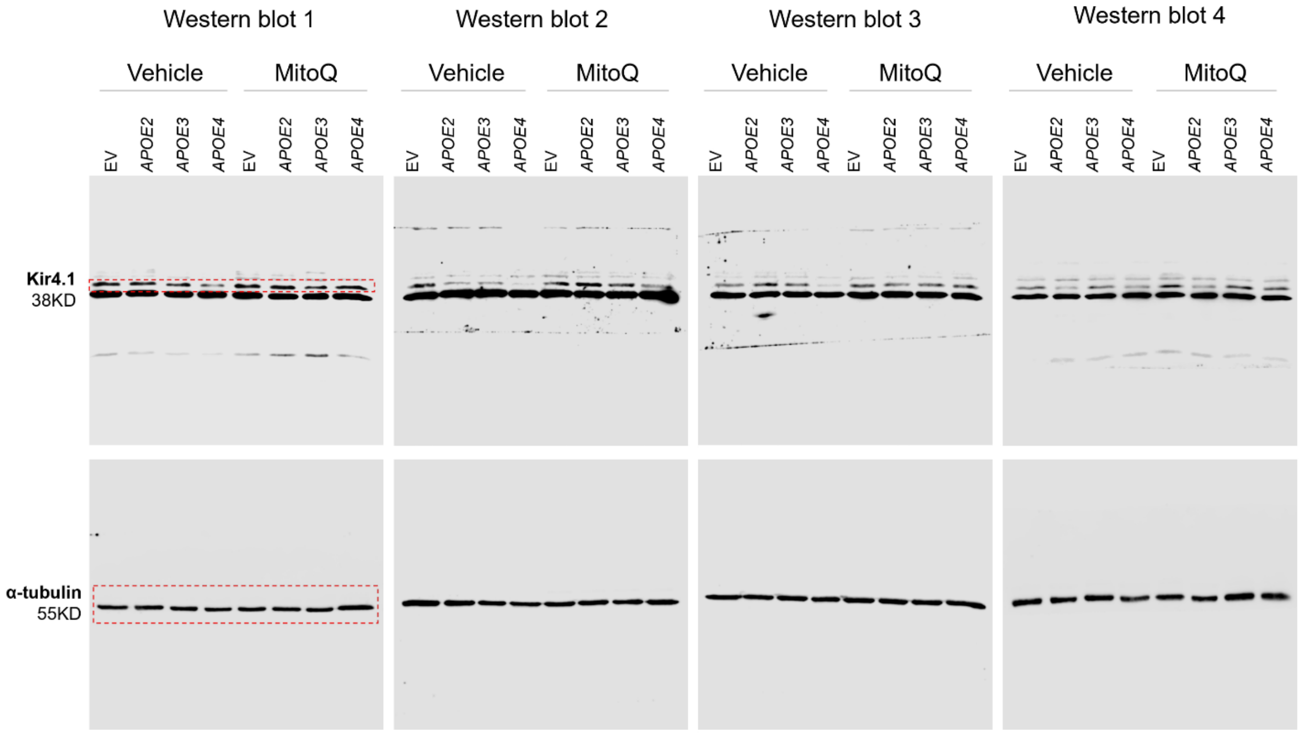

Western blots for Figure S9

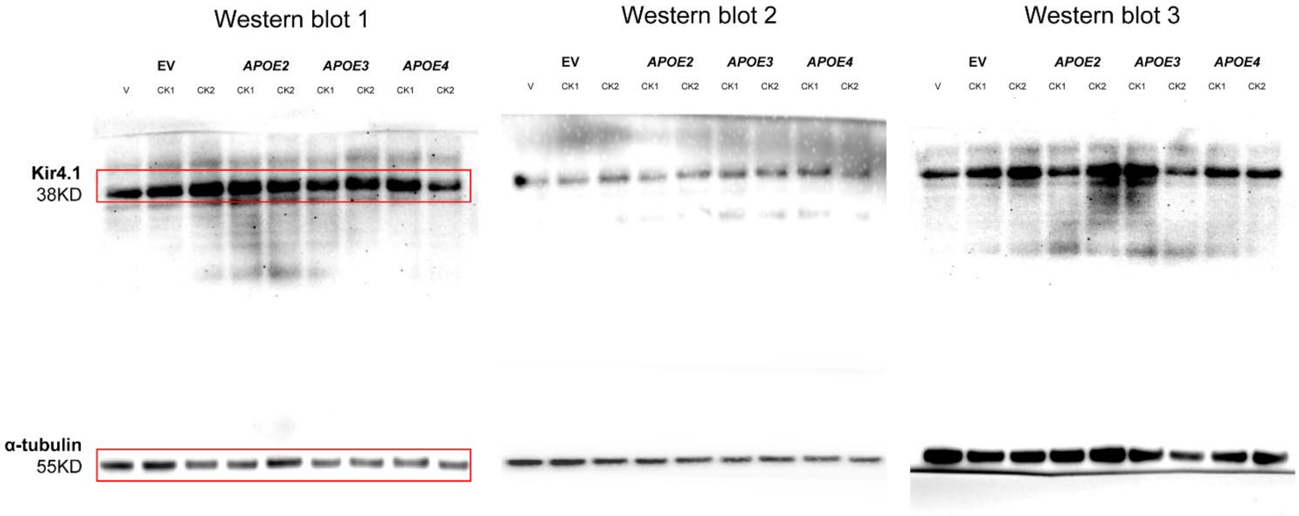

Supplement: Supplementary file 1 — Figure S1: glia70119‐sup‐0001‐FigureS1‐S9.docx. APOE4 leads to a reduction in AQP4 expression in the retina. (a) Immunofluorescence staining images of retinal sections stained with AQP4 from 12 to 13 months old APOE3 and APOE4 mice. AQP4 is expressed in the ganglion cell layer (GCL) and inner limiting membrane (ILM), consistent with its distribution to MC end‐feet. The APOE4 retinas showed a reduction in AQP4 expression compared to APOE3 retinas, suggesting APOE4 potentially impacts retinal water homeostasis. Scale = 20 μm (n: APOE3 = 3, APOE4 = 3. Inner plexiform layer (IPL), outer plexiform layer (OPL), outer nuclear layer (ONL)). (b) Quantification of AQP4 fluorescence intensity shows significantly reduced expression in APOE4 retinas (n: 11–12 images/group). Values are expressed as mean ± SEM. An unpaired t‐test was used for statistical analysis. **p < 0.01. Figure S2: APOE4 retinas show increased cholesterol accumulation independent of Kir4.1. (a) Ingenuity pathway analysis of retinal RNA‐seq data showing APOE4‐mediated activation of 3‐hydroxy‐3‐methyl‐glutaryl‐coenzyme A reductase (HMGCR), consistent with enhanced cholesterol biosynthesis. (b) Retinal agarose sections from APOE3 and APOE4 mice stained with Kir4.1 (green), and BODIPY (magenta). APOE4 retinas show increased BODIPY signal (shown in white arrows) but no colocalization with Kir4.1. Scale= 20 μm. (n: 3 mice/group). Figure S3: Confirmation of transfections. Representative images of rMC‐1 showing validation of transfection. rMC‐1 transfected with EV or human APOE2/APOE3/APOE4 were stained for each antibody: anti‐HA (for EV), total APOE, APOE3 and APOE4. Scale 20 μm (n: 3 independent experiments). Figure S4: APOE4 expression in rMC‐1 increases cholesterol while reducing Kir4.1. rMC‐1 transfected with EV/APOE2/APOE3/APOE4 and stained for Kir4.1 (green), and BODIPY (magenta). APOE4‐transfected cells show elevated BODIPY and reduced Kir4.1 expression, without colocalization between the two signals. Scale= [file GLIA-74-0-s001.pdf]
